# β-Blockers bearing hydroxyethylamine and hydroxyethylene as potential SARS-CoV-2 Mpro inhibitors: rational based design, *in silico*, *in vitro*, and SAR studies for lead optimization†

**DOI:** 10.1039/d1ra04820a

**Published:** 2021-11-03

**Authors:** Mohammed I. A. Hamed, Khaled M. Darwish, Raya Soltane, Amani Chrouda, Ahmed Mostafa, Noura M. Abo Shama, Sameh S. Elhady, Hamada S. Abulkhair, Ahmed E. Khodir, Ayman Abo Elmaaty, Ahmed A. Al-karmalawy

**Affiliations:** Department of Organic and Medicinal Chemistry, Faculty of Pharmacy, Fayoum University Fayoum 63514 Egypt mia06@fayoum.edu.eg; Department of Medicinal Chemistry, Faculty of Pharmacy, Suez Canal University Ismailia 41522 Egypt khaled_darwish@pharm.suez.edu.eg; Department of Basic Sciences, Adham University College, Umm Al-Qura University Saudi Arabia rasoltan@uqu.edu.sa; Faculty of Sciences, Tunis El Manar University Tunisia; Department of Chemistry, College of Science Al-Zulfi, Majmaah University Al-Majmaah 11952 Saudi Arabia amain.c@mu.edu.sa; Laboratory of Interfaces and Advanced Materials, Faculty of Sciences, Monastir University Monastir 5000 Tunisia; Institute of Analytical Sciences, UMR CNRS-UCBL-ENS 5280 5 Rue la Doua 69100 Villeurbanne CEDEX France; Center of Scientific Excellence for Influenza Viruses, National Research Centre Dokki Cairo 12622 Egypt ahmed_elsayed@daad-alumni.de noura.mahrous1995@gmail.com; Department of Natural Products, Faculty of Pharmacy, King Abdulaziz University Jeddah 21589 Saudi Arabia ssahmed@kau.edu.sa; Department of Pharmaceutical Organic Chemistry, Faculty of Pharmacy (Boys), Al-Azhar University Nasr City 11884 Cairo Egypt hamadaorganic@azhar.edu.eg; Department of Pharmaceutical Chemistry, Faculty of Pharmacy, Horus University-Egypt New Damietta 34518 Egypt akarmalawy@horus.edu.eg; Department of Pharmacology, Faculty of Pharmacy, Horus University-Egypt New Damietta 34518 Egypt akhodir@horus.edu.eg; Department of Medicinal Chemistry, Faculty of Pharmacy, Port Said University Port Said 42526 Egypt ayman.mohamed@pharm.psu.edu.eg

## Abstract

The global COVID-19 pandemic became more threatening especially after the introduction of the second and third waves with the current large expectations for a fourth one as well. This urged scientists to rapidly develop a new effective therapy to combat SARS-CoV-2. Based on the structures of β-adrenergic blockers having the same hydroxyethylamine and hydroxyethylene moieties present in the *HIV-1* protease inhibitors which were found previously to inhibit the replication of SARS-CoV, we suggested that they may decrease the SARS-CoV-2 entry into the host cell through their ability to decrease the activity of RAAS and ACE2 as well. Herein, molecular docking of twenty FDA-approved β-blockers was performed targeting SARS-CoV-2 Mpro. Results showed promising inhibitory activities especially for Carvedilol (CAR) and Nebivolol (NEB) members. Moreover, these two drugs together with Bisoprolol (BIS) as an example from the lower active ones were subjected to molecular dynamics simulations at 100 ns. Great stability across the whole 100 ns timeframe was observed for the top docked ligands, CAR and NEB, over BIS. Conformational analysis of the examined drugs and hydrogen bond investigation with the pocket's crucial residues confirm the great affinity and confinement of CAR and NEB within the Mpro binding site. Moreover, the binding-free energy analysis and residue-wise contribution analysis highlight the nature of ligand–protein interaction and provide guidance for lead development and optimization. Furthermore, the examined three drugs were tested for their *in vitro* inhibitory activities towards SARS-CoV-2. It is worth mentioning that NEB achieved the most potential anti-SARS-CoV-2 activity with an IC_50_ value of 0.030 μg ml^−1^. Besides, CAR was found to have a promising inhibitory activity with an IC_50_ of 0.350 μg ml^−1^. Also, the IC_50_ value of BIS was found to be as low as 15.917 μg ml^−1^. Finally, the SARS-CoV-2 Mpro assay was performed to evaluate and confirm the inhibitory effects of the tested compounds (BIS, CAR, and NEB) towards the SARS-CoV-2 Mpro enzyme. The obtained results showed very promising SARS-CoV-2 Mpro inhibitory activities of BIS, CAR, and NEB (IC_50_ = 118.50, 204.60, and 60.20 μg ml^−1^, respectively) compared to lopinavir (IC_50_ = 73.68 μg ml^−1^) as a reference standard.

## Abstract 2

Hydroxyethylamine and hydroxyethylene moieties of β-blockers exert potential SARS-CoV-2 inhibitory effects: rational-based design and *in silico*, *in vitro*, and SAR Studies.

## Graphical abstract

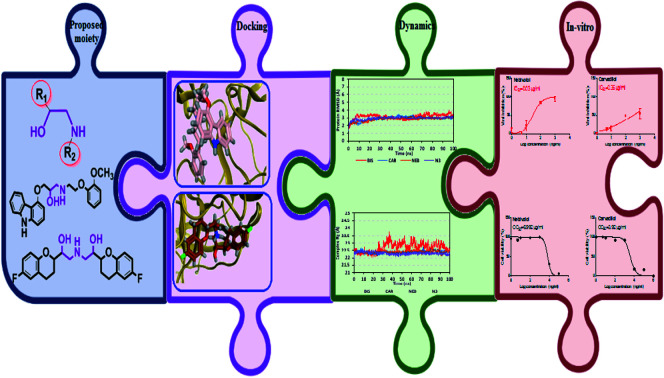

## 1. Introduction

The recent outbreak of novel corona virus pneumonia referred to as neo-coronary pneumonia caused by severe acute respiratory syndrome-related coronavirus 2 (SARS-CoV-2) in December 2019 raised global health concerns.^1^ Coronavirus disease (COVID-19) rapidly spread to other countries and by March 11^th^, the World Health Organization has announced COVID-19 as an international public health emergency.^2^ By June 9, 2021, approximately 174 738 762 patients were diagnosed with COVID-19, affecting 222 countries and territories around the world with a total death toll of 3 762 570.^3^ The virus is highly contagious, lethal especially for those suffering from other health issues, and with a bad impact on the economy and social life.^4^ Therefore, developing safe and effective anti-SARS-CoV-2 drugs is urgently needed.^5,6^

Corona viruses are classified into four genera (α, β, γ, and δ). Severe acute respiratory syndrome-related coronavirus (SARS-CoV), the Middle East respiratory syndrome coronavirus (MERS-CoV), and SARS-CoV-2 are β-coronaviruses.^7^ Analysis of the genome sequences of these three viruses has revealed that SARS-CoV-2 has a higher identity to SARS-CoV (89.1% nucleotide similarity) than to MERS-CoV.^8^ Moreover, the SARS-CoV-2 genome is a single-stranded positive-sense RNA of about 30 kb in length and contains at least six open reading frames (ORFs) that code for a minimum of 16 non-structural proteins and 4 structural proteins.^9^ The 229E gene encodes two polyproteins involved in releasing of functional polypeptides, and that are essential for viral replication and transcription. Besides, the protease responsible for the proteolytic processing is 3 chymotrypsin-like proteases of SARS-CoV-2 (3CLpro or Mpro), as it cleaves at least 11 sites on the polyproteins translated from the viral RNA.^10,11^ Given the relevance for the viral replication cycle, thus the viral protease (Mpro) has been proven as an attractive target in the development of inhibitors against coronaviruses.^12–19^

Currently, there is no single specific antiviral therapy for COVID-19 and the main treatments are only supportive.^20^ Drug repurposing is a strategy that adopted by several researchers to seek effective treatment in a short period.^21,22^ Besides, a virtual screening based on molecular docking emerges as an important tool for obtaining new antiviral molecules, where researchers can use this tool as a complementary approach prior to the synthesis of new promising compounds.^23–25^ While traditional methods of drug discovery could take years, the approach taken here is to search for possible medications for the SARS-CoV-2 through *in silico* screening (molecular docking and dynamics simulations) and *in vitro* studies of FDA-approved β-blockers towards the SARS-CoV-2 Mpro protein.

## 2. Rational of the work

**Fig. 1:**
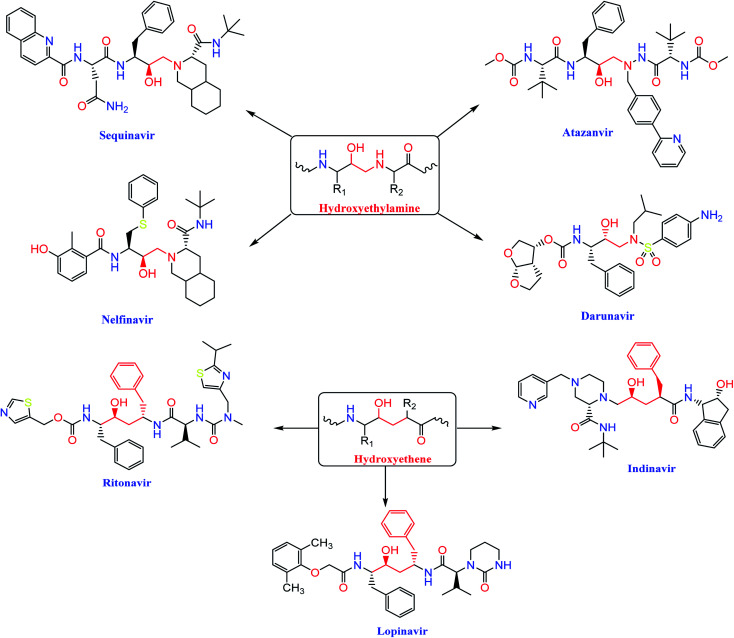
Structures of some *HIV-1* protease inhibitors containing the pharmacophoric hydroxyethylamine and hydroxyethylene moieties.

The *HIV-1* protease inhibitor, nelfinavir, strongly inhibits the replication of the SARS coronavirus (SARS-CoV). Nelfinavir inhibits the cytopathic effect induced by SARS-CoV infection.^26^ Similar to nelfinavir, ritonavir and lopinavir are recommended as protease inhibitors for the treatment of SARS and MERS, which have similar mechanisms of action as on HIV.^27^ Moreover, *HIV-1* protease inhibitors are structurally classified into two main categories: (i) hydroxyethylamine derivatives (sequinavir, atazanvir, nelfinavir, fosamperavir and darunavir) (ii) hydroxyethylene derivatives (rotinavir, indanavir and lopinavir) as depicted in Fig. 1.^28^

**Fig. 2:**
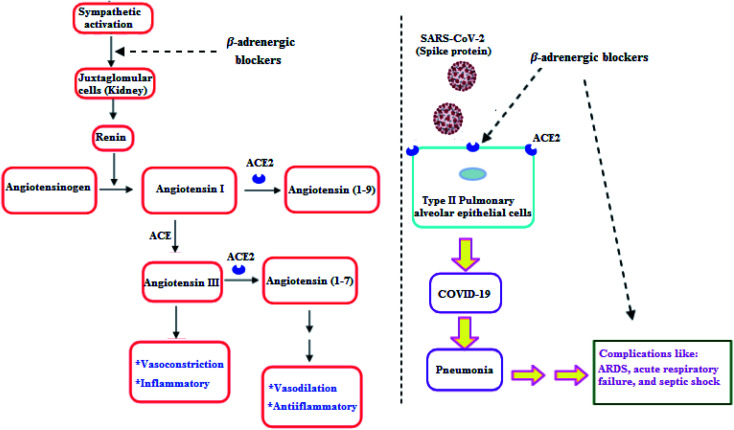
β-Adrenergic blockers effect on RAAS and SARS-CoV-2.^32^

Clearly, it is known that COVID-19 patients may exhibit pneumonia, and severe cases have complications like acute respiratory distress syndrome (ARDS), respiratory failure, and septic shock which are accompanied with high mortality rates.^29^ Furthermore, recent studies showed that β-adrenergic blockers reduced the mortality in septic shock patients.^30^ In addition, β-adrenergic blockers showed beneficial effects in ARDS and respiratory failure patients.^31^ Hence, β-adrenergic blockers by its inhibitory action on the sympathetic system, negatively regulate renin release by Juxtaglomerular (JG) cells in the kidney. So, a decrease in renin may reduce the activity in both arms of RAAS and may decrease ACE2, which may decrease the SARS-CoV-2 entry into the host cell (Fig. 2).^32^

**Fig. 3:**
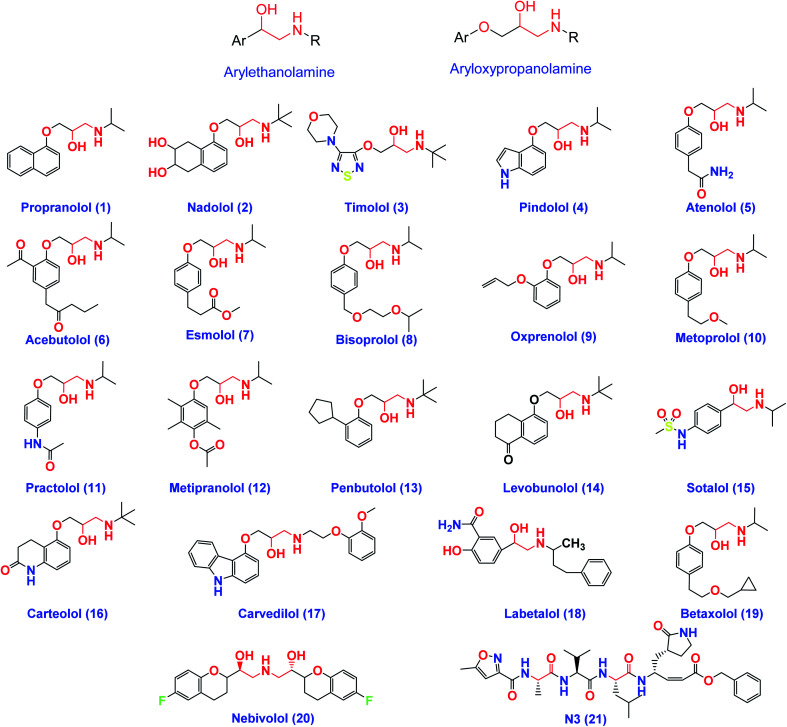
The chemical structures of some FDA-approved β-adrenergic blockers containing ethanolamine and/or aryloxypropanolamine moieties and the co-crystallized inhibitor ligand of SARS-CoV-2 Mpro (N3).

On the other hand, β-adrenergic blocking agents can be structurally classified into; arylethanolamine derivatives (Labetalol & Sotalol) which have structure similarity to *HIV-1* protease inhibitors and/or aryloxypropanolamine derivatives (Propranolol, Nadolol, Timolol, Atenolol, and Esmolol) as depicted in Fig. 3.^28^

So, the main objectives of this study are to determine the efficiency of most β-adrenergic blockers against SARS-CoV-2 using *in silico* and *in vitro* approaches and to purpose potential drugs which act against the catalytic domain of the SARS-CoV-2 Mpro. Besides, the studied β-adrenergic blockers can be used as lead compounds for further optimization in the future based on SAR studied attaining better activity against SARS-CoV-2 Mpro. Moreover, the use of approved β-adrenergic blockers drugs to treat COVID-19 has the advantage of assuring medical safety because these drugs have already been tested in animal models and undergone all the essential clinical trials. Additionally, the infrastructure to manufacture at large-scale is already in place.^33,34^

## 3. Methodology

### 3.1. Molecular docking

We conducted docking studies using MOE 2019 to examine the binding affinities of twenty β-adrenergic blockers (1–20) against SARS-CoV-2 Mpro. The co-crystallized inhibitor ligand (N3) was used as a reference standard.

### 3.2. Preparation of the tested β-adrenergic blockers

Structures of the tested compounds and the formal charges on atoms were checked by 2D depiction, subjected to energy minimization and the partial charges were automatically calculated. The tested compounds together with the co-crystallized ligand (N3) were imported in the same database and saved in the form of MDB file for the docking calculations with target protease as described earlier.^35,36^

### 3.3. Preparation of the target (SARS-CoV-2 Mpro)

Protein Data Bank was used to download the crystal structure of SARS-CoV-2 Mpro with (PDB code: 6LU7 and resolution: 2.16 Å).^37^ The crystal structure was protonated and hydrogen atoms were added with their standard 3D geometry, automatic correction for any errors in the atom's connection and type was applied, and potential fixation of the receptor and its atoms were done. Site Finder was applied for selection of the same active site of co-crystallized inhibitor using all default items and dummy atoms of the pocket were created.^38,39^

### 3.4. Docking of the tested β-adrenergic blockers to the viral Mpro binding site

Docking of the previously prepared database composed of the tested twenty β-adrenergic blockers (1–20) and the co-crystallized inhibitor N3 (21) was performed. The following methodology was applied: the file of the prepared active site was loaded, and general docking process was initiated. The program specifications were adjusted so that the docking site (dummy atoms), the placement methodology (triangle matcher) and the scoring methodology (London dG). Rigid receptor as refinement methodology and GBVI/WSA dG as the scoring methodology for selection of the best ten poses from one hundred different poses for each tested compound.^40,41^ The MDB file of the twenty-one ligands was loaded and general dock calculations were run automatically. The obtained poses were studied after completion, and the best ones having the best ligand–enzyme interactions and the most acceptable root mean square deviation (RMSD) values were selected and stored for energy calculations. At the beginning, a validation process was also performed for the target receptor by docking only the co-crystallized ligand and low RMSD values between docked and crystal conformations indicate a valid performance.^42,43^

### 3.5. Molecular dynamics simulations

In order to explore the stability and dynamic behaviour of the ligand–Mpro complex, promising β-adrenergic blockers were proceeded through a 100 ns all-atoms molecular dynamics (MD) simulation using GROMACS software.^44^ Generation of the ligand's topology and forcefield parameters was achieved using the CHARMM-General Force Field (CGenFF) program.^45^ Each ligand–Mpro complex was centered within a 3D-cubic box (100 × 100 × 100 Å), solvated using the TIP3P water model with a minimum of 10 Å marginal distance between each box side and protein. Ionizable residues were assigned for their respective standard ionization states under physiological conditions (pH = 7).^13,16,46^ Addition of sufficient numbers of K^+^ and Cl^−^ ions was done through the Monte-Carlo ion-placing method allowing efficient system neutralization.^47^ Finally, the prepared systems were simulated within periodic boundary conditions in order to eliminate any surface clashes. Both CHARMM36 force field and constant number of particles, pressure, and 303.15 K temperature (NPT) ensemble were considered.^48^ Following system construction, each of the prepared systems were subjected to one-step minimization and two-step equilibration stages. Such approach would ensure an efficient resolvement of any bad or even non-appropriate contacts between the componant of the system which may cause any system errors interruptions throughout the MD runs. Along both stages, a force constant of 1000 kJ mol^−1^ nm^−2^ was implemented to harmonically position-restrain all heavy atoms permitting a relevant preservation of the original protein folding.^49,50^ Steepest descent method was adopted throughout the minimization step for achieving a local energy minimum within the docked ligand–Mpro complexes permitting the resolve of any steric clashes or inappropriate geometry.

The minimization step was proceeded for 5000 steps (5 ps), followed by the thermal equilibration stage for ensuring a reasonable starting structure. Using the Berendsen thermostat for constant Number of particles, Volume, and Temperature (NVT) ensemble, the first equilibration stage was proceeded through a single-step protocol for 100 000 steps over a total 100 ps duration.^51^ As to follow, the second equilibration stage underwent for another 100 ps under constant Number of particles, Pressure (1 atm), and Temperature (NPT) ensemble using the Nose–Hoover thermostat and Parrinello-Rahman barostat for temperature control.^52^ The Verlet cut-off scheme was used for the non-bonded interactions (Coulomb's electrostatic and Lennard–Jones′ hydrophobic potentials) estimating a cut-off radius of 1 nm (10 Å). The Particle-Mesh Ewald (PME) algorithm was used for treating the long-range electrostatic interactions,^53^ whereas all covalent bonds including the hydrogen were constrained *via* the new LINear Constraint Solver (LINCS) algorithm.^54^ At this point, each system became minimized and well-equilibrated at the proper temperature (303.15 K), while being ready for the 100 ns duration molecular dynamics runs. Three MD simulation replica were carried out for each ligand–protein complex under NPT ensemble, Nose–Hoover thermostat and Parrinello-Rahman barostat for maintaining a canonical ensemble, and at an integration time step of 2 fs with no restrictions. The Verlet cut-off scheme (cut-off radius of 10 Å) was adopted for treating the long-range interactions.

Data analysis was performed using the GROMACS tools *via* several build-in trajectory tools including the root-mean-square deviation (RMSD), radius of gyration (*R*_g_), and RMS-Fluctuations (RMSF) to determine the molecular complex stability/validity in terms of MD performance, flexibility, and conformation.^55^ All MD data are reported with standard deviation of the multiple runs. For better estimation of the protein flexibility, the difference RMSF (ΔRMSF) was estimated for each ligand–bound protein relative to the SARS-CoV-2 Mpro apo state (PDB code: 6Y84; atomic resolution 1.39 Å), where ΔRMSF = apo RMSF – holo RMSF. The same previous preparation, minimization, equilibration, and 100 ns all-atom MD simulation production were applied to the Mpro apo state, except no ligand preparation was performed. The Hydrogen Bond Analysis and Distance Calculation Tool, within Visual Molecular Dynamics ver.1.9.3 software (VMD; University of Illinois, Urbana-Champaign, USA) were utilized. The cut-off values for hydrogen bond (donor–H⋯acceptor; DH–A) distance and angle were assigned at 3.0 Å and 20°, respectively.^56,57^ These tools would allow respective estimation of the number/frequency of ligand–Mpro intermolecular hydrogen bonding as well as monitoring the distance changes between the specified ligand/protein atoms (hydrogen bond donor and acceptors) over the whole simulation periods. Finally, the binding-free energy was estimated by the Molecular Mechanics/Poisson–Boltzmann Surface Area (MM/PBSA) calculation using the *g_mmpbsa* module within GROMACS. The MM/PBSA calculations provided more insights regarding the magnitude of ligand–protein affinity, the nature of interaction, in addition to the residue-wise contributions within the binding-free energy calculations.^58^ Important MM/PBSA parameters for polar/solvation calculations were set at solvent dielectric constant (80 pdie), solute dielectric constant (2 pdie), radius of solvent probe (1.40 Å), and reference vacuum (1 vdie). Concerning SASA apolar solvation; the radius of SASA solvent probe, offset constant, and solvent surface tension were set at 1.40 Å, 3.8493 kJ mol^−1^, and 0.0227 kJ mol^−1^ Å^−2^, respectively. Finally, parameters for continuum-integral based model were set as solvent probe radius 1.25 Å, bulk solvent density (0.0334 Å^−3^), and 200 for numbers of quadrature points per Å^2^. All MM/PBSA calculations were applied on representative frames at defined intervals using the GROMACS “*gmx trjconv*” followed by the “*gmx trjcat*” command scripts. For representing the ligand–protein conformational analysis across specific timeframes, the Schrödinger™ Pymol™ graphical software ver. 2.0.6 was used.^59^

### 3.6. *In vitro* studies

#### 3.6.1. MTT cytotoxicity assay

This assay was conducted in order to calculate the minimum concentrations of the tested compounds that cause 50% toxicity (CC_50_) to the cells. In the beginning, ddH_2_O with 10% DMSO was used to dissolve the examined compounds which were diluted with Dulbecco's Modified Eagle's Medium (DMEM) during work. The MTT method with minor modifications was performed using VERO-E6 cells which are suitable for the virus propagation to be used in other experiments. Simply, VERO-E6 cells were kept in 96 well-plates at 37 °C in 5% CO_2_ for 24 h to be cultivated. The tested compounds were diluted with DMEM in HA plate in triplicates as mentioned before and then poured onto the prepared cells after washing twice by sterile 1× phosphate buffer saline (PBS). 24 h later, the cell monolayers were washed three times with sterile 1× PBS after removal of the supernatant followed by the addition of the MTT solution into each individual well (20 μl of 5 mg ml^−1^ stock solution) which was kept at 37 °C for 4 h. The formed formazan crystals were dissolved using an acidified isopropanol (200 μl) and the absorbance of their solutions were recorded through a multi-well plate reader (*λ*_max_ = 540 nm) against a reference wavelength (*λ*_max_ = 620 nm). Finally, the cytotoxicity % of the tested compounds compared to the control cells (untreated cells) was calculated as follow:
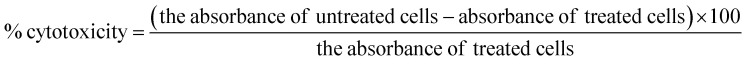


#### 3.6.2. Inhibitory concentration 50 (IC_50_) determination

The Vero-E6 cells (2.4 × 104) were kept overnight at 37 °C in 5% CO_2_ inside 96-well tissue culture plates. 1× PBS solution was used to wash the cell monolayers for only one time which were then treated with different serial dilutions of the examined compounds together with a fixed dilution from the virus (hCoV-19/Egypt/NRC-03/2020 (Accession Number on GSAID: EPI_ISL_430820)) following TCID_50_ test and kept at RT for 1 h before starting incubation. Also, the cell monolayers were subjected to DMEM (100 μl) with different concentrations of the test samples and virus and left at 37 °C for 72 h in a 5% CO_2_. Then, 4% paraformaldehyde (100 μl) was used for cell fixation (2 h) followed by the staining step with 0.1% crystal violet in distilled H_2_O (50 μl) at RT for 15 min. Absolute CH_3_OH (100 μl) was added to dissolve the crystal violet dye per well to measure the optical density of the produced color using Anthos Zenyth 200rt plate reader at 570 nm.^60^ The IC_50_ value for each tested compound which is corresponding to its minimum concentration required to reduce the virus infectivity by 50% in comparison to the virus control was calculated.

#### 3.6.3. SARS-CoV-2 main protease assay

The *3CL Protease Assay Kit* was designed to measure the Mpro activity for screening and profiling applications, in a homogeneous assay with no time-consuming washing steps. The kit came in a convenient 96-well format, with purified 3CL Protease, fluorogenic substrate, and 3CL Protease assay buffer for 100 enzyme reactions. 3CL inhibitor GC376 was also included as a positive control. The applied methodology and protocol were described in detail in the ESI (SI1†). Therefore, the SARS-CoV-2 Mpro assay was performed to evaluate the inhibitory effects of the tested compounds (BIS, CAR, and NEB) towards the SARS-CoV-2 Mpro enzyme.

## 4. Results and discussion

### 4.1. Molecular docking

**Fig. 4:**
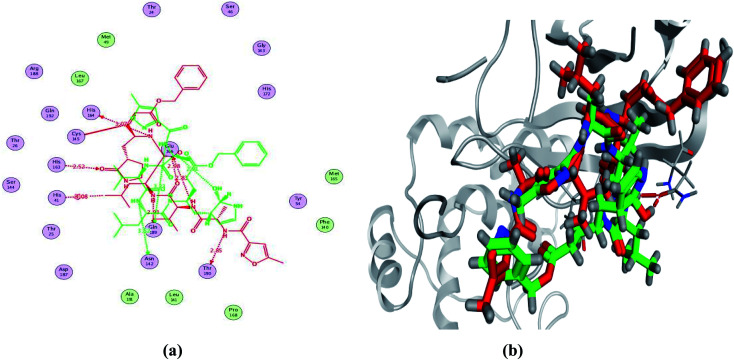
2D diagram (a) and 3D representation (b) of the superimposition of the co-crystallized (red) and the docking pose (green) of N3 in the SARS-CoV-2 Mpro binding site.

In order to study the binding characteristics of the β-adrenergic blockers with the binding site of Mpro, molecular docking studies were performed by Molecular Operating Environment (MOE, 2019.10) software. The SARS-CoV-2 Mpro has a Cys–His catalytic dyad, and the substrate-binding site is located in a cleft between domains I and II. The N3 inhibitor is fitted inside the substrate-binding pocket of SARS-CoV-2 Mpro showing asymmetric unit containing only one polypeptide. The X-ray crystallographic structure of Mpro (PDB ID: 6LU7)^37^ was downloaded from the Protein Data Bank (PDB).^38^ The downloaded protein is co-crystalized with the inhibitor N3. Molecular docking protocol was initially validated by re-docking of the co-crystalized ligand (N3) in its binding site of Mpro (Fig. 4). The simulation successfully reproduced the binding pattern of the co-crystalized ligand in the Mpro binding site with energy score of −9.61 kcal mol^−1^, and with an RMSD of 1.90 Å, between the docked pose and the co-crystalized ligand. The validation step results indicate the suitability of the used molecular docking protocol for the molecular docking study of the β-adrenergic blockers in the binding sites of SARS-CoV-2 Mpro.

Molecular docking simulation of propranolol 1, nadolol 2, timolol 3, pindolol 4, atenolol 5, acebutolol 6, esmolol 7, bisoprolol 8, oxprenolol 9, metoprolol 10, practolol 11, metipranolol 12, penbutolol 13, levobunolol 14, sotalol 15, carteolol 16, carvedilol 17, labetalol 18, betaxolol 19, nebivolol 20, and N3 inhibitor 21 into the SARS-CoV-2 Mpro active site was done. They got stabilized at the N3-binding site of Mpro by variable several electrostatic bonds (Table 1). The order of strength of binding was as follows: N3 (21, docked) > carvedilol 17 > nebivolol 20 > acebutolol 6 > labetalol 18 > esmolol 7 > bisoprolol 8 > penbutolol 13 > betaxolol 19 > timolol 3 > metipranolol 12 > carteolol 16 > levobunolol 14 > metoprolol 10 > nadolol 2 > sotalol 15 > oxprenolol 9 > atenolol 5 > propranolol 1 > practolol 11 > pindolol 4.

**Table 1:** Receptor interactions and binding energies of the identified β-adrenergic blockers and N3 inhibitor into the N3 inhibitor binding site of SARS-CoV-2 Mpro

No.	β-Adrenergic blockers	*S* a kcal mol^−1^	RMSD_Refine^b^	Interacting amino acids	Distance Å
1	Propranolol	−6.04	1.97	Gln189/H–donor	3.20
				Glu166/H–acceptor	3.29
				His164/H–donor	3.21
				His41/H–pi	3.59
				Asn142/H–pi	4.37
2	Nadolol	−6.40	1.49	His163/H–acceptor	2.87
				Gln189/H–donor	3.98
				Met49/H–donor	3.58
				Asn142/H–pi	3.68
3	Timolol	−6.61	0.94	—	—
4	Pindolol	−5.75	1.83	Gln189/H–donor	3.15
				Asn142/H–pi	4.14
5	Atenolol	−5.90	1.78	Glu166/H–acceptor	2.97
				His164/H–donor	3.38
				Met49/H–donor	4.44
				His41/H–pi	3.85
				Asn142/H–pi	3.68
6	Acebutolol	−6.94	0.97	His41/H–pi	4.00
7	Esmolol	−6.77	1.50	Glu166/H–donor	3.10
				Glu166/ionic	3.35
				Glu166/ionic	3.57
				His41/H–pi	4.10
**8**	**Bisoprolol**	**−6.67**	**1.40**	**Asn142/H–donor**	**3.37**
				**Glu166/H–donor**	**2.86**
9	Oxprenolol	−6.28	1.82	Met49/H–donor	4.29
				His41/H–pi	3.76
				His163/H–pi	4.27
10	Metoprolol	−6.40	2.11	Met49/H–donor	3.59
				His41/H–pi	4.53
11	Practolol	−5.93	0.54	His163/H–acceptor	3.03
				His41/H–acceptor	2.96
12	Metipranolol	−6.50	1.12	Gln189/H–donor	3.30
				Gly143/H–acceptor	3.25
13	Penbutolol	−6.66	1.99	Gln189/H–donor	3.32
14	Levobunolol	−6.46	1.67	Met49/H–donor	3.91
				His163/H–acceptor	3.42
15	Sotalol	−6.28	0.90	His163/H–acceptor	3.05
				Met165/H–pi	4.1
16	Carteolol	−6.47	0.77	Gln189/pi–H	4.17
**17**	**Carvedilol**	**−7.33**	**1.93**	**Gln189/H–donor**	**3.41**
				**His41/H–acceptor**	**3.15**
				**His41/cation–pi**	**4.48**
				**Asn142/pi–H**	**4.15**
18	Labetalol	−6.78	1.96	Gln189/H–donor	3.07
19	Betaxolol	−6.63	1.95	Glu166/H–donor	3.02
**20**	**Nebivolol**	**−7.08**	**0.96**	**His164/H–donor**	**3.28**
				**Met49/H–donor**	**3.17**
				**Met165/pi–H**	**3.59**
**21**	**N3**	**−9.61**	**1.90**	**Gln189/H–donor**	**3.23**
				**Asn142/H–donor**	**3.52**
				**Glu166/H–donor**	**2.91**

a *S*: Score of a compound into the binding pocket of protein using London dG scoring.

b RMSD_Refine: Root-Mean-Squared-Deviation (RMSD) between the predicted pose (after refinement) and the crystal structure (before refinement).

Many poses were obtained with better binding modes and interactions inside the receptor pocket. The poses with the best scores (related to the stability of the pose) and RMSD values (related to the closeness of the selected pose to the original ligand position inside the receptor pocket) were selected. The detailed binding modes of the docked N3 (21) and all the tested β-adrenergic blockers (1–20) were represented in Table 1. Furthermore, all of their 2D and 3D binding interactions were depicted in the ESI (Fig. SI1†).

The results of docking studies revealed that Carvedilol 17 (CAR) and Nebivolol 20 (NEB) have the best binding affinities against SARS-CoV-2 Mpro with binding free energies of −7.33 and −7.08 kcal mol^−1^, respectively (Table 1). These energy values were more near to that of the docked N3 inhibitor (binding energy = −9.61 kcal mol^−1^). In addition, the docked N3 moiety occupied the branched pocket of Mpro forming three hydrogen bonds with Glu-166, Gln-189, and Asn-142. On the other hand, CAR showed the formation of two H-bonds with His-41 and Gln-189, cation–pi interaction with His-41, and pi–H interaction with Asn-142. Moreover, NEB exhibited two hydrogen bonds with Met-49 and His-164, and another pi–H interaction with Met-165. Also, Bisoprolol 8 (BIS) showed (binding energy = −6.6734 kcal mol^−1^) through the formation of two H-bonds with Glu-166 and Asn-142. The 3D binding interactions and 3D protein positioning of the docked N3 and the best selected β-adrenergic blockers were represented in Table 2.

**Table 2:** 3D view of the binding interactions and the 3D positioning between the most promising β-adrenergic blockers (BIS, CAR, and NEB) and N3 within SARS-CoV-2 Mpro pocket (PDB: 6LU7) compared to the N3 inhibitor (Docked). Red and gray dashed lines refer to hydrogen bonds and hydrophobic interactions, respectively

Drug	3D interactions	3D positioning
BIS (8)	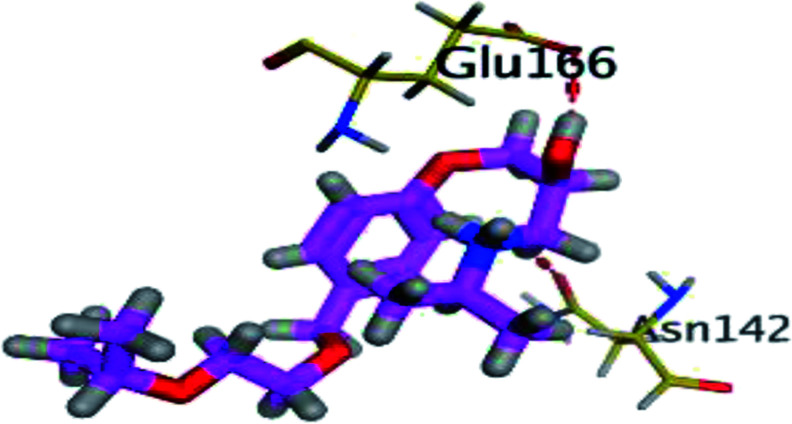	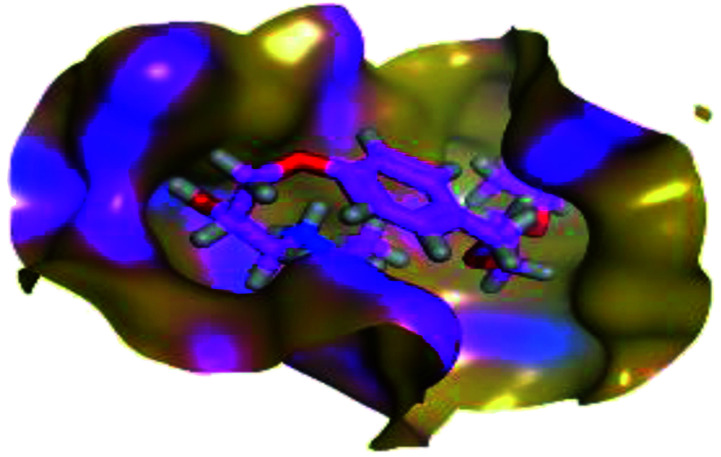
CAR (17)	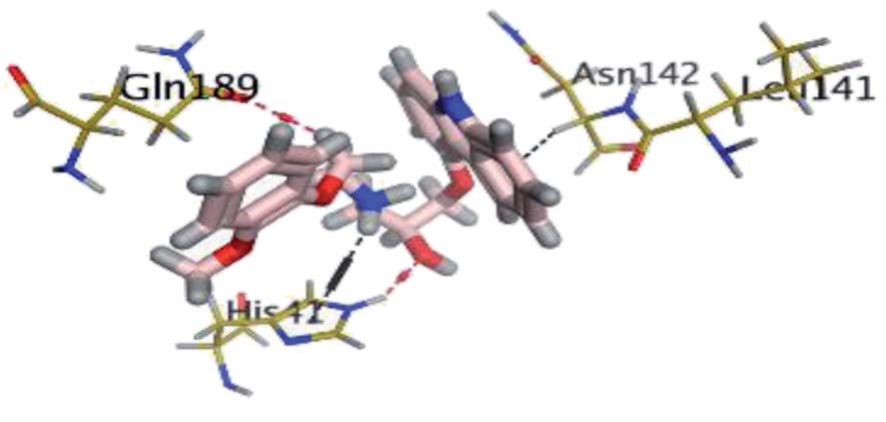	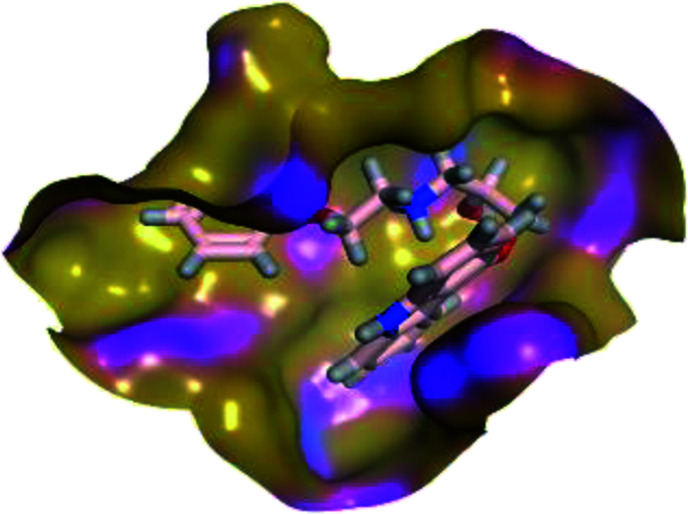
NEB (20)	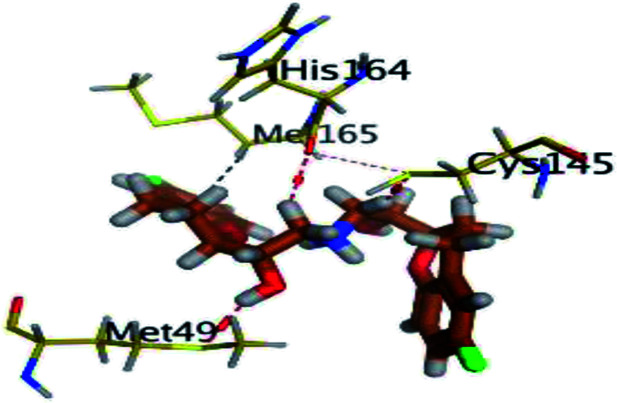	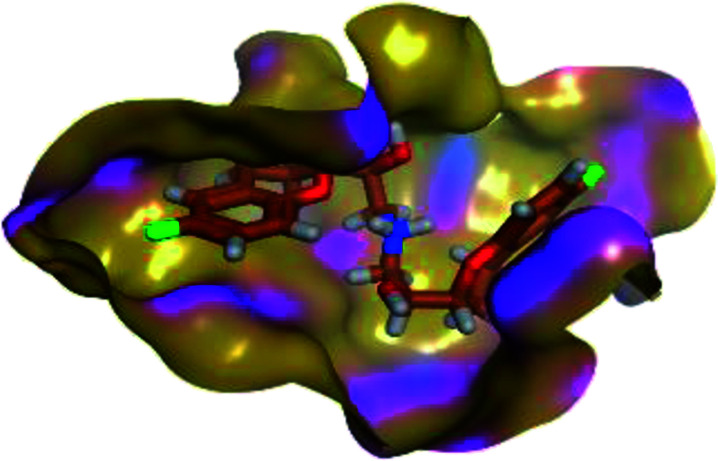
N3 (21)	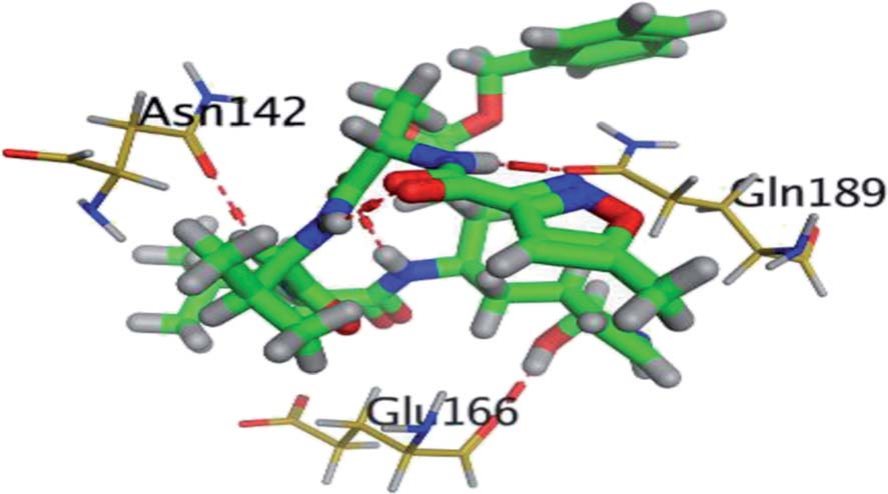	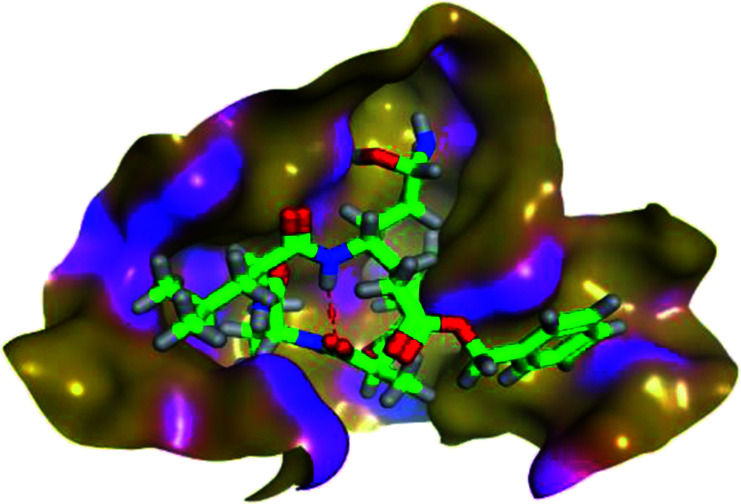

### 4.2. Molecular dynamics simulations

#### 4.2.1. MD simulation analysis of promising SARS-CoV-2 Mpro target inhibitors

Being an effective tool for investigating the relative stability of ligand-target complex as well as their respective dynamic behavior, MD simulation studies were performed. The latter computational tool is considered particularly beneficial for exploring the conformation space of ligand–target complex being more efficiently than other *in silico* tools including molecular docking and mechanics energy minimization approaches for just static image analysis.^61^ The top docked poses with relevant ligand–Mpro interactions and affinity were subjected to 100 ns all-atom MD simulation. Therefore, the docked binding complex of BIS, CAR, and NEB within the SARS-CoV-2 Mpro canonical binding site were chosen for understanding their respective ligand/target conformational alterations across the interaction course.

##### 4.2.1.1. Global stability analysis of ligand–protein complexes

**Fig. 5:**
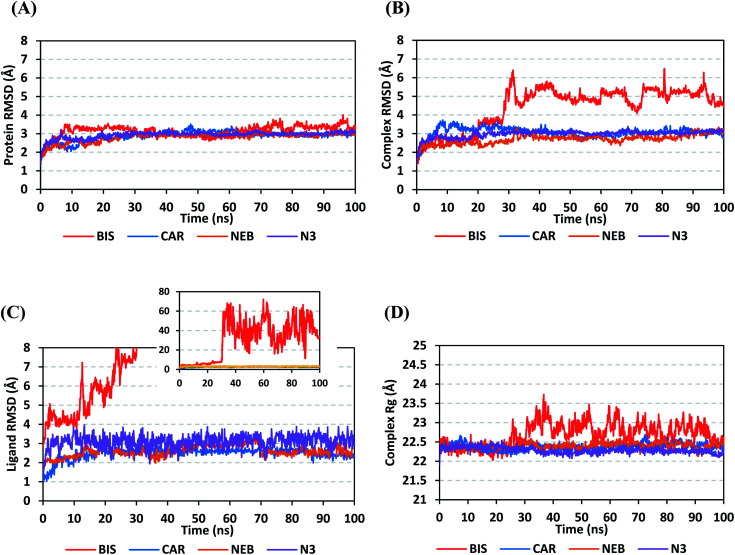
Stability analysis of generated MD trajectories for β-blocker drugs and reference ligand in complex with SARS-CoV-2 Mpro protein along 100 ns all-atom MD simulation. (A) Protein C-α RMSD; (B) complex backbone RMSD; (C) sole ligand backbone RMSD with a zoomed-out chart representing the whole BIS ligand RMSD; (D) complex *R*_g_ trajectories, across simulation time (ns).

Throughout the 100 ns all-atom MD runs, the examined β-blocker agents illustrated significant global stability within the target's canonical binding site as being confirmed through the monitored RMSD and radius of gyration (*R*_g_) trajectories. Generally, RMSD estimates the molecular deviation of a particular ligand relative to a designated original/reference structure. Such analytical tool would provide a good indication for the ligand-target stability and the adopted MD simulation protocol was valid. Target's instability and significant conformational alterations are associated with high RMSD trajectories.^62^ On the other hand, high values of complex RMSD would correlate to limited ligand-target affinity where the ligand is unable to be confined within target's canonical binding site along the simulation periods.^63^ The estimated RMSD deviations for Mpro proteins, in reference to their respective C-alpha atoms (C-α RMSD), depicted an overall typical behavior for MD simulations (Fig. 5A). Over the initial frames, the protein's C-α RMSD tones increases as a result of constrain release at the beginning of MD simulation runs. Following the first ten ns of the MD runs, steady protein's C-α RMSD trajectories were obtained for more than half of the simulation run time (>50 ns), except for minimal fluctuation for BIS-bound protein near the MD end. This protein's dynamic behaviour indicates the successful convergence of the target proteins since the C-α RMSD were levelled off at 3.18 ± 0.25 Å, 2.98 ± 0.13 Å, 2.93 ± 0.11 Å, and 2.99 ± 0.08 Å for BIS, CAR, NEB, and N3, respectively, across the trajectory plateau and till the end of MD simulation course. Notably, the N3-bound protein managed to exhibit the steadiest C-α RMSD tones, exhibiting the lowest standard deviation value after the equilibration was attained. The above depicted protein's C-α RMSD tones also infer that successful system minimization, relaxation, and thermal equilibration stages has been adopted before the MD production step and thus, no further extension of the MD simulation beyond the 100 ns period was needed.

For gaining more insights concerning the ligand's confinement within the canonical binding site of Mpro across the MD run, the RMSD fluctuations were monitored for the combined ligand–protein complex in reference to the protein backbone initial frame (Fig. 5B). Despite limited fluctuations, the binary complexes of Mpro with CAR, NEB, and reference ligand (N3) managed to reach their respective dynamic equilibrium illustrating backbone RMSD plateau, beyond the 30 ns, indicating sufficient complex stability. Despite the differential backbone RMSD tones at the initial MD simulation frames, the three complexes managed to converge along the last 100 ns reaching to a final RMSD around 2.97 ± 0.22 Å. Achieving an early equilibration and steady backbone RMSD trajectories with the lowest average value (2.70 ± 0.25 Å), the NEB–Mpro complex illustrated significant ligand accommodation within the protein pocket as compared to CAR (3.07 ± 0.28 Å) and reference ligand, N3 (2.98 ± 0.18 Å). On the other hand, the BIS–Mpro complex trajectories were just steady till the first 30 ns MD simulation run, where afterwards strong fluctuations were depicted indicating dramatic ligand shift or even escape form the target's pocket. As an additional descriptor for ligand's retainment within the target pocket, the sole ligand RMSDs, relative to the reference protein backbone frame, were monitored along the MD simulation runs (Fig. 5C). Lower average RMSD trajectories were assigned to the stable β-blocker ligands, CAR (2.49 ± 0.29 Å) and NEB (2.62 ± 0.29 Å), as compared to N3 (3.09 ± 0.35 Å). This could be reasoned to the higher extent of N3 structural flexibility incorporating more rotatable bonds as compared to the stable β-blockers. In agreement with the above BIS–Mpro complex RMSD trajectories, very high ligand RMSD trajectories were assigned for BIS (average 30.49 ± 16.29 Å) ensuring its escape from the target binding site following the 30 ns of MD run. It worth mentioning that protein RMSD trajectories were within 1.5-fold those of their respective ligands, except for BIS, the thing that further confirms the successful convergence of the stable three ligand–protein complexes inferring the suitability of 100 ns simulation timeframe needing no further MD extension.

Further stability analysis of the investigated ligand–Mpro complex was done *via* monitoring the respective complex *R*_g_ trajectories (Fig. 5D). The *R*_g_ stability parameter allows the exploration of molecular compactness and rigidity along the MD runs. Typically, *R*_g_ is defined as root-mean-square distance of the mass-weighted atom group relative to respective common center of mass. Therefore, the overall dimensional changes and structural alterations of a small or macromolecule could be explored across the MD simulation time course.^64^ Under valid MD simulation, the molecular structure stability is associated with the *R*_g_ tones levelling off at an equilibration plateau around an average value. Except for BIS, steady complex *R*_g_ trajectories were assigned for both β-blocker agents (CAR and NEB) and reference N3. Showing average *R*_g_ tones of 22.38 ± 0.09 Å, 22.40 ± 0.08 Å, and 22.29 ± 0.08 Å for CAR, NEB, and N3, respectively, have ensured significant compactness and rigidity of these ligand–bound protein complex as favoured intra- or inter-molecular interactions around this time frame. Notably, the top three stable ligand–Mpro complexes converge around similar *R*_g_ value (22.46 ± 0.11 Å), at the MD simulation run ends, confirming the significant comparable complex compactness and stability.

##### 4.2.1.2. Local protein flexibility and fluctuation of target's residues

A local stability analysis was performed through estimating the RMSF stability validation parameter which able to highlight the residue-wise contribution within the target protein stability. Typically, RMSF provides valuable evaluation of the target's residues dynamic behaviour represented as both fluctuation and flexibility, through estimating the average deviation of each protein's amino acid in relation its respective reference position across time.^65^ Within the presented manuscript, the difference root-mean-square fluctuation (ΔRMSF) was a better estimation of the protein local flexibility being the RMSF difference for each ligand-bound protein relative to the SARS-CoV-2 Mpro apo state (ΔRMSF = apo RMSF – holo RMSF). A ΔRMSF cut-off value of 0.30 Å was relevant for estimating the significant alterations within the protein's structural movements meaning that amino acids with ΔRMSF above 0.30 were considered of limited mobility.^56^ Investigating the RMSF trajectories essentially execute for a trajectory region considered stable. Based on the above protein's C-α RMSD analysis, the target is of significant conformational stability along the 100 ns MD simulations for all systems the thing that allowed performing the C-α RMSF calculations for the whole MD trajectories.

**Fig. 6:**
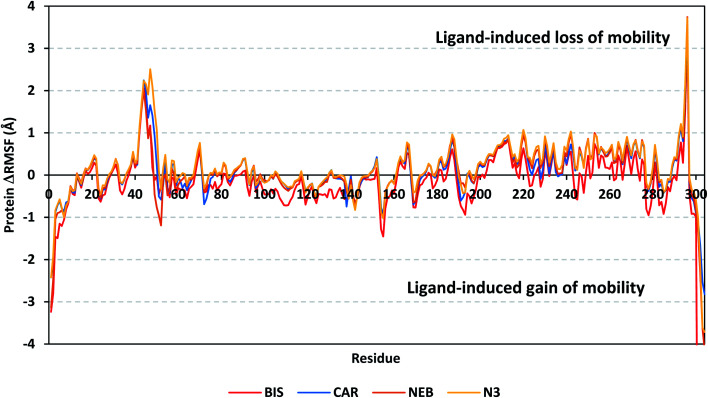
Analysis of ΔRMSF trajectories *versus* residue number for Mpro protein, in complex with both β-blocker compounds and reference ligand, throughout the whole MD simulation window. The ΔRMSF values, in reference to protein C-α atoms, were estimated considering independent MD simulation of Mpro apo-state (PDB code: 6Y84; atomic resolution 1.39 Å) against the holo ones being complexed with the investigated β-blocker ligands or reference N3. The ΔRMSF trajectories are represented as a function of residue number (residues 1-to-306).

Throughout the ΔRMSF analysis, the free terminals residues and respective vicinal residues showed typical fluctuation pattern with the highest negative ΔRMSF values in comparison to the core residues (Fig. 6). Higher fluctuation patterns were depicted for the residues of each ligand–protein complex at the regions down towards the Mpro N-terminus as compared to those located near the carboxylic end. Notably, the terminal flexible residues are at regions located >15 Å from the protein's canonical binding site. The latter infers to the ability the active site to accommodate bulkier ligands. Several distinct residue ranges including 41–48, 162–167, 185–188, and 203–296, illustrated significant immobility possessing an average ΔRMSF more than 0.30 Å threshold. Notably, the residue range 290–296 being vicinal to the protein's N-terminal showed one of the highest immobility profiles (ΔRMSF up to 3.74 ± 0.08 Å). This was short for a couple of residues at the BIS–bound Mpro protein. Such dynamic behavior confers significant influence of ligand's binding upon the stability of these N-terminal vicinal residues. Concerning comparative protein's local stability, lower ΔRMSF trends were assigned for BIS-bound Mpro residues relative to those of the other β-blockers and reference ligand. This was recognized across several ranges of protein's residues, most notably for 41–52 and 145–155 residue ranges. The presented ΔRMSF findings confer with the high stability of CAR- and NEB-bound proteins being comparable to that of N3 coming in great concordance with the previously discussed RMSD and *R*_g_ data.

Regarding the specific flexibility of the pocket residues, almost all canonical pocket residues depicted significant immobility with ΔRMSF above the cut-off mobility threshold 0.30 Å (Table 3). Residues lining the S1′ subsite showed significant mobility with the lowest of all ΔRMSF values (lots of negative values) the thing that infers the great mobility indices of such residues. However, only the catalytic His-41 showed limited flexibility only for CAR, NEB, and reference ligand (ΔRMSF = 0.31 ± 0.08 Å, 0.33 ± 0.06 Å, and 0.37 ± 0.06 Å, respectively), while that for BIS was at the marginal cut-off (0.29 ± 0.11 Å). The other catalytic residue, Cys-145, exhibited significant mobility with all ΔRMSF being of negative values. This indicates the significant role of the ligand-His-41 hydrogen bond pair over that of Cys-145 for stabilizing the ligand–protein complex. This was in great concordance with our previous study investigating promising natural scalaranes sesterterpenes isolated from the Red Sea marine sponge *Hyrtios erectus* as promising inhibitors of SARS-CoV-2 Mpro target.^46^ Moving towards the S1 subsite, significant high ΔRMSF values were depicted across the four ligand–bound proteins regarding a couple of pocket lining residues, His-163 and Glu-166. Significant immobility for the Glu-166 ensures the reported data in current literature suggesting the crucial role of S1 subsite Glu-166 residues for stabilizing several drug-like and peptidomimetic ligands at the Mpro active site.^13,16,46,66–70^ Finally, almost all residues lining the S1 sub-pocket and few comprising the S3 one (Met-165, Leu-167, and Gln-189) showed high trends of significant immobility. Values of ΔRMSF for these latter residues were significantly high ranging from 0.32 ± 0.18 Å and up to 1.53 ± 0.43 Å. It is worth mentioning that this immobility trade was higher in NEB as compared to other β-blocker and even the reference ligand, N3.

**Table 3:** Estimated ΔRMSF^a^ values of ligand–Mpro proteins along the whole MD simulation

Binding site subsite	Residue	BIS–Mpro protein	CAR–Mpro protein	NEB–Mpro protein	N3–Mpro protein
S1′	His-41	0.29 ± 0.11	*0.31 ± 0.08*	*0.33 ± 0.06*	*0.37 ± 0.06*
	Gly-143	−0.54 ± 0.13	−0.54 ± 0.08	−0.53 ± 0.05	−0.48 ± 0.28
	Ser-144	−0.28 ± 0.07	−0.24 ± 0.12	−0.20 ± 0.07	−0.17 ± 0.28
	Cys-145	−0.14 ± 0.07	−0.15 ± 0.04	−0.09 ± 0.03	−0.06 ± 0.64
S1	Phe-140	−0.02 ± 0.19	−0.03 ± 0.21	−0.11 ± 0.74	−0.24 ± 0.64
	Leu-141	−0.49 ± 0.21	−0.48 ± 0.21	−0.47 ± 0.62	−0.60 ± 0.27
	Asn-142	−0.66 ± 0.24	−0.72 ± 0.12	−0.74 ± 0.64	−0.83 ± 0.55
	His-163	*0.32 ± 0.12*	*0.38 ± 0.09*	*0.43 ± 0.04*	*0.44 ± 0.03*
	Glu-166	*0.52 ± 0.32*	*0.65 ± 0.14*	*0.73 ± 0.15*	*0.77 ± 0.27*
S2	Met-49	−0.12 ± 0.40	*0.93 ± 1.55*	*0.46 ± 0.43*	*1.53 ± 0.43*
	Tyr-54	*0.39 ± 0.25*	0.02 ± 0.10	*0.33 ± 0.26*	*0.48 ± 0.26*
	His-164	0.21 ± 0.17	*0.31 ± 0.03*	*0.32 ± 0.18*	0.29 ± 0.16
	Asp-187	*0.61 ± 0.15*	*0.84 ± 0.22*	*0.87 ± 0.05*	*0.96 ± 0.06*
	Arg-188	*0.36 ± 0.09*	*0.66 ± 0.38*	*0.68 ± 0.08*	*0.85 ± 0.35*
S3	Met-165	0.15 ± 0.23	0.27 ± 0.03	*0.34 ± 0.16*	*0.35 ± 0.29*
	Leu-167	*0.49 ± 0.33*	*0.61 ± 0.24*	*0.63 ± 0.17*	*0.72 ± 0.15*
	Gln-189	−0.11 ± 0.19	0.19 ± 0.56	*0.36 ± 0.39*	*0.40 ± 0.43*
	Thr-190	−0.52 ± 0.26	−0.23 ± 0.48	0.08 ± 0.56	−0.04 ± 0.19
	Gln-192	−0.82 ± 0.43	−0.53 ± 0.29	−0.19 ± 0.68	−0.43 ± 0.22

a Relative difference root-mean-square fluctuation (ΔRMSF) ± standard deviation was estimated for each ligand-associated Mpro protein relative to the SARS-CoV-2 Mpro apo-state (PDB code: 6Y84; atomic resolution 1.39 Å). Residues showing significant immobility (ΔRMSF > 0.30 Å) are written in bold and values are in italic.

Several vicinal residues for the S1′ subsite, and less extend for S2 subsite, depicted significant rigidity. These immobile residues include, Pro-39, Val-42 to Asp-48, Phe-185, and Val-186 inferring the stability of ligands within these two respective protein subsites. Again, NEB-bound Mpro showed the highest ΔRMSF values regarding these S1′ and S2 vicinal residues. In brief, the provided ΔRMSF findings illustrated the key role S2 and S3 amino acids, S1 Glu-166, S1′ catalytic His-41, as well as vicinal residues for stabilizing β-blocker compounds and N3 within the Mpro canonical pocket. All these came in high concordance with the above presented *R*_g_ behaviours as well as findings from RMSD. Thus, ΔRMSF trajectories positively add to suggested sustained stability and compactness of the β-blocker-Mpro investigated complexes, particularly for CAR and NEB rather than BIS, across the all-atoms MD simulations.

##### 4.2.1.3. Conformational analysis of ligand–protein complex across selected trajectories

**Fig. 7:**
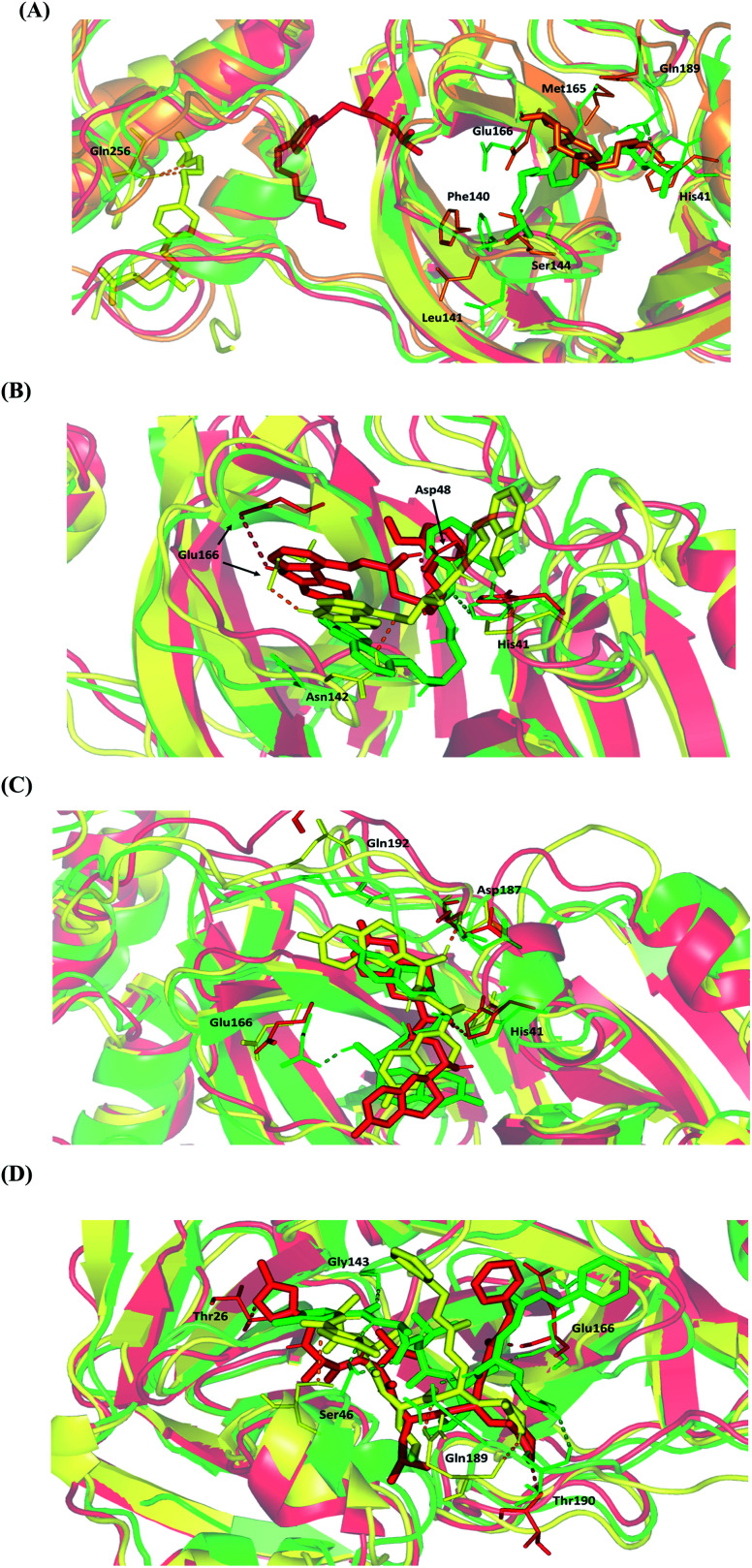
Conformational analysis of ligand–protein complex at SARS-CoV-2 Mpro binding site across selected trajectories. (A) BIS; (B) CAR; (C) NEB; (D) N3 reference ligand. Protein is represented in green, orange, yellow, and red cartoon 3D-representation corresponding to 0 ns, 20 ns, 60 ns, and/or 100 ns extracted frames, respectively. The key binding residues (lines), ligands (sticks), and hydrophilic interactions (hydrogen bonding as dashed lines), are all presented in colors corresponding to their respective extracted frame.

For further analysis of key conformational alterations across the MD simulation timeframe, selected frames of each ligand–protein system were extracted and minimized to a 0.001 kcal.mol^−1^.A^−2^ gradient using MOE system preparation package. The comparative conformational analysis was conducted at stable RMSD trajectories for the ligand–protein complex in reference to respective backbone. Notably, a significant molecular drift was depicted for BIS across the MD simulation run (Fig. 7A). At 20 ns frame, characteristic features were depicted including the loss of ligand's relevant polar interaction at the S3 subsite between the ligand's terminal 3^ry^ amine and Gln-189 sidechain. Additionally, a significant drift of the ligand's aliphatic arm, together with its aromatic center ring, out of the Mpro S1 subsite was also depicted at 20 ns frame. Both losing the initial docking hydrogen bond pair with Gln-189 and the drift of towards the solvent side mediating the escape of BIS from the Mpro canonical pocket throughout the forthcoming trajectories. At the 60 ns frame, BIS was settled at a surface pocket at the domain-III of Mpro at a distance of more than 40 Å from the catalytic substrate-binding site. The latter correlates with the extremely large ligand RMSD trajectories around this MD simulation time course (Fig. 5C). Stability of the BIS-protein complex at 60 ns was facilitated through double polar interactions with Gln-256 mediated through the two oxygen atoms incorporated within the ligand's aliphatic arm with its terminal 3^ry^ amine arm directed towards the solvent side. The significant drift of BIS was continued as the MD run proceeded beyond the 60 ns allowing the ligand to be anchored at another surface pocket at the Mpro domain-III at the end of MD simulation run. Depicting no significant polar interactions at the last BIS orientation suggests its poor stability at this site inferring its continuous drift over an extended MD simulation run.

Moving towards CAR–protein complex, this 9*H*-carbazole-based β-blocker agent showed great confinement within the Mpro catalytic site along the MD simulation runs (Fig. 7B). The ligand showed slight orientation shift towards the S1 and S2 subsites of the target's canonical pocket. The initial hydrogen bond pair between the catechol scaffold and S1′ catalytic His-41 sidechain was lost as the ligand terminal arm showed significant rotation around its aliphatic linker bonds. The latter dynamic behaviour was primary driven by the significant flexibility of CAR at its ethyl amino propan-2-ol central linker. Interestingly, the ligand's inherited flexibility was also beneficial for stabilizing the ligand within the active site due to bringing the ligand's polar/hydrophobic functionalities close to crucial pocket residues. At 60 ns MD time course, stability of CAR at the protein pocket was driven through polar interactions between the 9*H*-carbazole-4-yloxy terminal arm with S1 subsite residues: Glu-166 sidechain Asn-142 main chain. Interaction with the pocket's crucial ligand-anchoring residue, Glu-166, have been reported essential for the stability of several ligands at Mpro pocket.^13,16,46,66–70^ The important role of Glu166 for stabilizing the CAR-Mpro complex further extended till the end of the MD simulation were the Glu166:9*H*-carbazole hydrogen bond pair was maintained at 100 ns. This final adopted conformation by CAR was further stabilized through polar interaction with the pocket vicinal residue, Asp-48, *via* the ligand's free hydroxyl group. Such relevant interaction aided in the smooth transition of the CAR's terminal catechol ring towards the S2 subsite.

The last investigated β-blocker, NEB, showed great confinement within the target active site, even more profound than CAR. The two dihydro-2*H*-chromen moieties, at the far ends of the ligand structure, showed limited position shift at the Mpro pocket (Fig. 7C). This limited orientation change could be reasoned for shorter central linker causing allowing lower inherited flexibility for the two terminal aromatic rings as compared to CAR. Additionally, some of the most crucial pocket residues served as polar anchors for NEB to be perfectly settled within the target hydrophobic pocket. Despite the loss of initial polar interaction between S1 Glu-166 sidechain and ligand's free hydroxyl group, stronger polar interaction was established with the S2 subsite lining residues (Asp-187) showing the ligand's other OH at proximity. At the end of MD simulation (100 ns), the S1′ catalytic His-41 served as the ligand's anchor instead of Asp187 with the same ligand's hydroxyl group. It worth noting that the orientation of His-41 was not altered significantly across the investigated MD frames as compared to Glu-166 or even Asp-187. The latter can highlight the significant role of His-41 within ligand–protein complex stability through offering a transient polar anchoring site for NEB anchoring across the MD simulation run. Concerning the terminal ring orientation of CAR at S3 subsite, the hydrophilic residue Gln-192 showed closeness towards the ligand's terminal fluoro substitution at the initial docking pose. Such favoured orientation could favour relevant hydrogen bonding with the Gln-192 sidechain acting as hydrogen-bond doner. However, such proximity was lost at later MD simulation windows (60 ns and 100 ns) where the hydrophilic amino acid moves far from the ligand's terminal. All above conformational analysis could rationalize the obtained low average RMSD trajectories for both the NEB–protein complex and its sole components.

Finally, the N3–Mpro complex showed less significant orientation shift at the target pocket similarly to the most stably bounded β-blockers (Fig. 7D). This could explain the steadiest RMSD trajectories being obtained for the N3–Mpro binary complex (Fig. 5). Notably, several hydrogen bond interactions of the initial docking pose were conserved between the Mpro key pocket residues and N3 functionality. Both hydrogen pairs between the ligand's N3 proline scaffold and S3 Thr-190 mainchain as well as the ligand's phenyl ester arm and S1 Glu-166 mainchain were found conserved at the end of the MD simulation run. On the other hand, the initial hydrophilic contacts with Ser-46 sidechain and S1′ Gly-143 main chain were lost against the modified amino acid within the N3 structure at 100 ns. Nevertheless, only the Ser-46 hydrogen pair was maintained across the 60 ns frame. Losing these polar interactions at significant could be the reason for the profound anchoring of ligand's oxazole ring to a deeper extent towards the S1′ vicinal cleft. The latter orientation was further stabilized through a polar hydrogen bond pair interaction between the N3-incorporated unnatural amino acid and mainchain of Thr-26 at 100 ns of MD simulation run.

##### 4.2.1.4. Hydrogen bond analysis and evolution of ligand–protein intermolecular distances

**Fig. 8:**
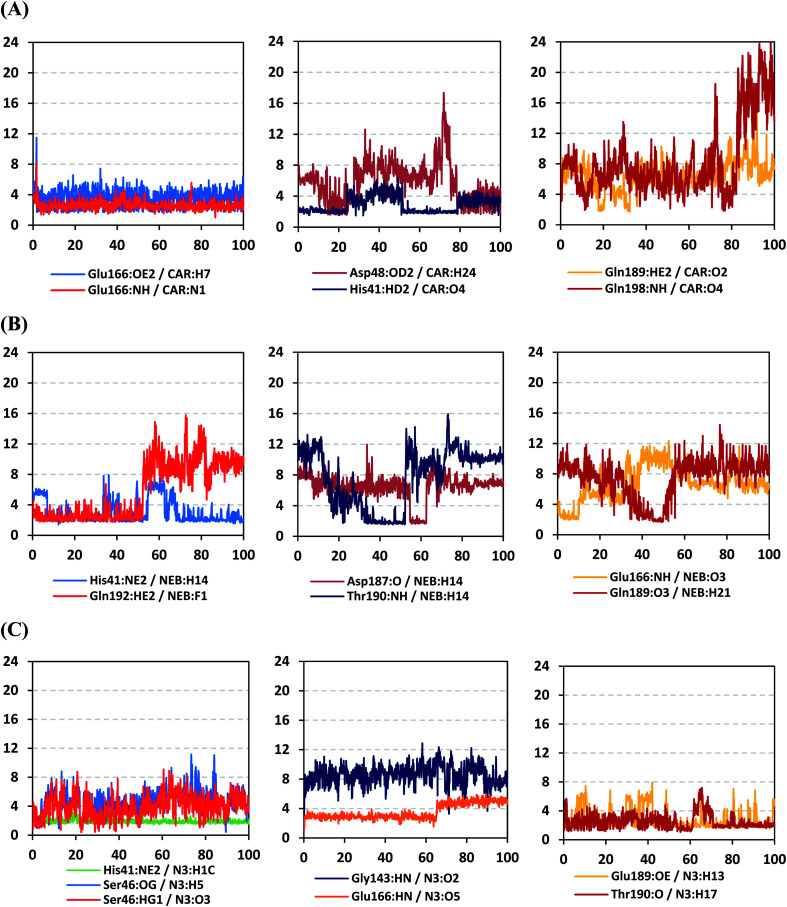
Time-evolution of hydrogen bond donor–acceptor distances for selected β-blocker agents and reference inhibitor with Mpro key binding residues across 100 ns MD simulation run. (A) CAR; (B) NEB; (C) N3 reference ligand. The *X*- and *Y*-axes correlates to the MD simulation time in nanoseconds and apparent hydrogen bond (donor–*H*⋯acceptor; DH–A) distances in Å, respectively.

For better understanding of the observed ligand conformational changes, investigating the time evolution of hydrogen bond donor–acceptor distances between Mpro residues and ligands was considered crucial. Monitoring these distances across MD simulation times would comprehend the important role of particular Mpro residue as well as residue-wise specificity for the stability of a ligand in target's pocket. Notably, more than 20 hydrogen bond pairs were depicted for CAR-protein complex including the key pocket residues responsible for CAR recognition and anchoring at initial docking pose as well as in the above conformational analysis (Fig. 8A). The longest timeline was assigned for both the S1 Glu-166 and S1′ catalytic His-41 sidechains at frequencies of 63.75 ± 0.45% and 42.04 ± 0.33%, respectively. The hydrogen donor–acceptor (DH–A) distances between CAR-9*H*-carbazole and Glu-166 were monitored for both the residue's mainchain-NH and sidechain-OE2. Steadier and lower average DH–A distances were assigned for the Glu-166 sidechain rather than its mainchain (2.58 ± 0.50 Å *versus* 3.61 ± 1.04 Å). The latter observation infers the superior contribution of Glu166 mainchain over its sidechain for stabilizing the CAR–protein complex. Moreover, this favoured hydrogen bond pair (CAR-9*H*-carbazole: Glu-166-NH) evolved steady after the first 5 ns and continued till the end of the MD simulation run. The latter dynamic behaviour agrees with the above conformational analysis regarding the important role of Glu-166 for stabilizing CAR following the initial docking pose as well as around both 60 ns and 100 ns.

Concerning the DH–A distances between His-41 sidechain-HD1 and the free methoxy of the CAR's catechol ring, limited fluctuations and very small distances were depicted across certain MD timeframes. Only at the time courses around 0–20 ns and 50–80 ns showed very close DH–A average distances (2.10 ± 0.23 Å and 1.99 ± 0.16 Å, respectively) inferring a highly significant hydrogen bond interaction and the crucial role of His-41 for CAR anchoring during these timeframes. It worth noting that His-41 DH–A distances at the rest of the MD run never exceeded the 7 Å distance inferring great confinement of the CAR 9*H*-carbazole terminal arm within the active site. Monitoring the DH–A distance for pocket vicinal residue, Asp-48, with CAR catechol oxygens illustrated limited contribution for ligand anchoring since average distances were below 4 just following 80 ns (3.65 ± 0.99 Å) and around 20 ns (3.25 ± 1.20 Å). This was correlated to minimal occupancy (9.25 ± 0.23%) conferring its ligand-stabilizing role to be at the end of the MD run as being illustrated through the above conformational analysis. Finally, few S2 and pocket vicinal residues showed negligible lifetime and high DH–A distances across the MD simulation run including Thr-26 (0.60 ± 1.24%), Ser-46 (0.30 ± 0.67%), Phe-140 (0.10 ± 1.03%), Asp-187 (0.60 ± 0.45%), and Gln198 (1.60 ± 0.77%).

Monitoring the DH–A distances for the NEB–Mpro complex has highlighted the crucial role of several pocket residues for the stability of this chromone-based ligand at the active site. The time evolution of His-41-NE2 (N atom of the His side chain imidazole ring) distances with the free hydroxy group of NEB has illustrated favoured hydrogen bonding across several MD simulation time courses (Fig. 8B). The latter DH–A bond distance showed close proximity around and favoured hydrogen bonding following the first 5 ns of the MD simulation. Despite limited fluctuation around 40 ns and 60 ns, steady trajectories were depicted till the end of MD run at average DH–A distances of 2.22 ± 0.50 Å. This presented dynamic behaviour allowed the His-41-NE2: NEB-H14 bond distance to show high significant hydrogen bonding frequency (59.45 ± 1.31%) which could reason the limited orientation of His-41 and its crucial anchoring role of NEB as being described above at the conformational analysis. This could explain the profound activity of this compound that could be able to hamper the catalytic activity of SARS-CoV-2 Mpro enzyme. Another steady trajectory was assigned for the DH–A bond distance between the S1′ Gln-192 sidechain and terminal fluoro substitution at the ligand’ terminal scaffold. With a short DH–A distances around 2.88 ± 0.70 Å, the Gln-192-HE2 (H of the amide functional group of the Gln sidechain): NEB-F1 (Fluoro atoms of the NEB structure) was considered significant for more than half the MD simulation run (0 ns to 57 ns) exhibiting high frequency of 75.25 ± 0.79%.

Examining the hydrogen bonds with moderate lifetime, both the S2 Asp-187 (4.23 ± 0.53%) and S3 Thr-190 (9.87 ± 1.34%) residue have showed interesting findings through their orientations towards the free hydroxyl moiety of the NEB's linker. The DH–A bond distance for S3 Thr-190 (2.06 ± 0.69 Å) illustrated favoured hydrogen bonding only around 40 ns and before the 60 ns MD simulation runs. Subsequently, the bond distance significantly increased inferring the loss of hydrogen bonding with NEB till the end of the MD run at 100 ns. On the other hand, the mainchain of S2 Asp-187 showed proximity of a distance towards the ligand's linker favouring hydrogen bond just around 60 ns (1.95 ± 1.89 Å). The latter behaviour was confirmed within the above conformal analysis of NEB at 60 ns MD time frame. Based on both observation it is suggested that both Asp-187 and Thr-190 were responsible for NEB–Mpro stability for nearly 30 ns of the MD simulation run. Their combined hydrogen bonding contributions were suggested significant for anchoring NEB at the S2/S3 subsites despite their sole moderate hydrogen bonding frequency. At last, several pocket residues including the S1 Glu-166 and S3 Gln-189 mainchains have exhibited small hydrogen bond contributions (∼0.50 ± 2.31%). Limited favoured DH–A bond distances were depicted for Glu-166 with the free hydroxyl functionality of the NEB's linker across the initial frames of the MD simulation (0-to-10 ns). The latter correlates with the above conformational analysis, where Glu-166 polar interaction was lost at 60 ns and MD simulation end.

Regarding the DH–A analysis for the N3–Mpro complex, a lower number of polar contacts were depicted as compared to CAR (Fig. 8C). Adopting the previously mentioned DH–A angle and distance cut-offs has revealed only 14 hydrogen bonds as compared to 25 and 27 for CAR and NEB, respectively. Serving as HD with its sidechain, the S1′ catalytic His-41 illustrated moderate occupancy (15.30 ± 0.66%) with the steadiest tones along the entire MD simulation window. Being kept at average DH–A distance (1.91 ± 0.27 Å) from ligand's H1C-atom confer the significant role of His-41 for N3-pocket stability. The crucial S1 subsite residue, Glu-166, depicted favored DH–A distances for hydrogen bonding being mediated *via* its mainchain-NH, particularly at the initial 65 ns trajectories. Comparable hydrogen bond occupancy (16.12 ± 0.41%) was depicted for Glu-166 and His-41 residues. Both previous findings confirm the important role of His-41 sidechain and Glu-166 mainchain for anchoring both proteinomimetic and non-peptide based small ligands at Mpro catalytic pocket. Notably, bonding with Glu-166 was lost (reaching up at 6.00 ± 1.02 Å) after 65 ns the thing that could be correlated to the significant elevation of complex *R*_g_ trajectories following the same 65 ns simulation window. It could be concluded that compactness of N3-proytein complex was highly associated with the ligand–protein mutual interactions, as *R*_g_ increase could be correlated to a more weakened ligand–protein interactions.

Other pocket residues, such as the sidechains of Glu-189 and Thr-190, showed interrupted DH–A distances with several maxima across various time intervals. Nonetheless, the S3 Glu-189 residue exhibited the highest hydrogen bond frequency (24.50 ± 1.00%) inferring the significant contribution of Glu-189 for N3 anchoring within S3 subsite. Finally, poor hydrogen bonding was illustrated for the vicinal Ser-46 mainchain (7.06 ± 0.63%) and Gly-143 mainchain (0.91 ± 0.43%) across the 100 ns MD run. These DH–A distances were rapidly lost/elevated following few initial MD frames. It worth mentioning that losing or weakening of several hydrogen bonding being previously identified relevant through initial docking analysis have been correlated to significant conformation/orientation changes within the binding pocket. This came in great agreement with the previously described conformational analysis.

#### 4.2.2. Binding-free energy calculations

The binding-free energy calculation was performed to understand the nature of ligand–protein interaction as well as obtain more detailed information concerning the individual ligand contribution.^71^ In this regard, the MM/PBSA calculation was implemented for binding-free energy estimation, where higher negative binding energy explains more ligand affinity towards its respective target pocket.^58^ The MM/PBSA is considered of comparable accuracy to the Free-Energy Perturbation approaches, yet with much smaller computational expenses.^58^ Using the SASA-only model of the free-binding energy calculation as well as the single trajectory approach, representative frames were extracted/saved from the whole MD simulation trajectories to be used for calculating each energy term across the three MD simulation runs and their average values. Adopting the calculation across the 100 ns MD simulation time course was rationalized by the rapidly attained equilibration/convergence of the complex RMSD trajectories following few initial MD frames (Fig. 5B).

To our delight, both CAR and NEB as β-blocker agents bound to SARS-CoV-2 Mpro target have depicted significant free-binding and affinity to target's pocket (Table 4). Dissecting the obtained binding-free energy into its contributing energy terms, the van der Waal interactions showed superior contribution within the free-binding energy calculation of N3–protein complex as compared to Coulomb's electrostatic potential energy. This was opposite for both β-blocker agents where electrostatic potential energy was nearly the double that of the van der Waal hydrophobic interactions. This came in good reason since both β-blocker agents showed higher number and frequency of hydrogen bonding with the target's pocket as compared to those of N3. Based on current literature, the Mpro pocket is considered of more hydrophobic in nature for being deep, less solvent exposed, and with conserved hydrophobic pocket lining residues. However, the ability of CAR and NEB to establish favoured strong polar interactions with the pocket's key residues allow them to be deeply anchored and attaining significant pocket specificity. This was obvious through the previously described conformational and hydrogen bonding analysis. It worth mentioning that the total non-polar interactions (Δ*G*_van der Waal_ plus Δ*G*_SASA_) were lower for β-blockers as compared to those of N3 would have been directly related to the pocket's large surface area. Being hydrophobic and with large surface area, the Mpro binding site could favour higher non-polar interactions with N3 where the latter attained a more extended conformation within the target's pocket. Finally, similar binding pattern was depicted for CAR and NEB across the docking and MD simulation study. Both CAR and NEB exhibited more preferential free-binding energy as compared to N3 at Mpro binding site, the thing that confirms their superiority target affinity over N3 within the preliminary docking analysis. It worth noting that the profound free-binding energy of the top-stable β-blockers (CAR and NEB) correlates well with the obtained initial docking score ranking (Table 1).

**Table 4:** Average of total binding-free energies and individual energy term (Δ*G* ± SD; across three MD simulation replicas) concerning the promising β-blocker compounds and reference N3 at Mpro protein binding sites

Energy (kJ mol^−1^ ± SD)	Mpro complex		
	CAR	NEB	N3
van der Waal	−137.81 ± 8.03	−103.37 ± 10.77	−283.51 ± 10.36
Electrostatic	−234.86 ± 12.87	−211.02 ± 21.73	−46.70 ± 9.34
Solvation; polar	185.27 ± 15.32	125.01 ± 26.78	188.93 ± 14.27
Solvation; SASA	−16.26 ± 0.94	−13.36 ± 1.15	−23.53 ± 0.87
Binding energy	−203.65 ± 10.00	−202.74 ± 12.57	−164.81 ± 14.87

**Fig. 9:**
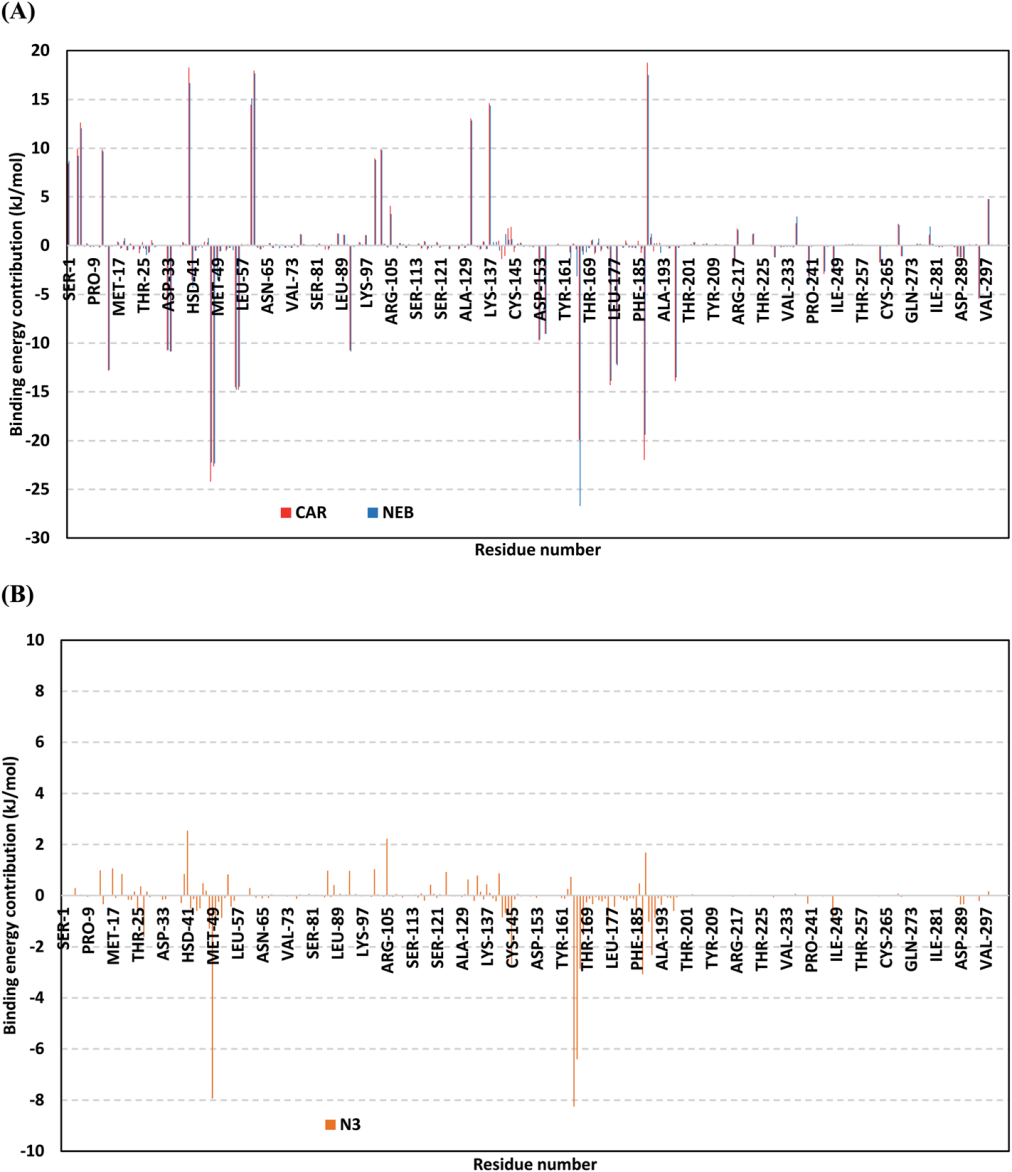
Binding-free energy decomposition illustrating the contribution of the protein target residues within the ligand/Mpro complexes binding-free energy calculation. (A) Blocker agents, CAR and NEB; (B) N3. This function was calculated through the *g_mmpbsa* tool in GROMACS.

For gaining more insights regarding ligand–residues interactions, the binding-free energy decomposition within the *g_mmpbsa* module was utilized to identify the key residues involved within the obtained binding free energies.^58^ Interestingly, similar residue-wise energy contribution patterns were assigned for both the β-blocker agents (Fig. 9A). This was of no surprise since both ligands depicted comparable energy terms as well as total binding-free energy values. Residues of the S2 subsites and their vicinal residues showed significantly high residue-binding energy contributions with values up to two-digit kJ mol^−1^. Residues such as; Glu-47, Asp-48, Glu-55, Asp-56, Asp-187, and Asp-197 illustrated very high energy contributions ranging from −14.49 ± 0.95 kJ mol^−1^ up to −24.22 ± 1.33 kJ mol^−1^. The high contribution for Asp-187 and Asp-48 came in great concordance with the previously described conformational and hydrogen bond analysis inferring the crucial role of these residues within ligand anchoring. Contribution of the key S1 sub-pocket residues was only assigned high for Glu-166 exhibiting high residue-associated energy contribution of −19.90 ± 7.55 kJ mol^−1^ and −26.72 ± 1.82 kJ mol^−1^ for CAR and NEB, respectively. Insignificant energy contributions were depicted for the S1 subsite residues, while as the residues of the S1′ sub-pocket showed only single contribution for the catalytic dyad, His-41. It is worth mentioning that the His-41 energy terms were lower than those of S2 and its vicinal residues implying the superior role of the S2 residues in stabilizing β-blocker agents at Mpro pocket.

Moving forwards for the reference inhibitor, the residue-wise energy contribution for the Mpro protein in complex with N3 showed the significant values for crucial pocket residues (Fig. 9B). However, such contributions were at much lower extent for the number of the involved residues and magnitude of energy contribution as compared to those with any of the top-stable β-blocker agents. The highest residue-contribution share was depicted among the S2 and S3 Mpro subsites, where the S2 Met-49 and S3 Met-165 residues assigned for the N3 respective greatest contributions at a Δ*G*_binding_ of −7.92 ± 0.14 kJ mol^−1^ and −8.25 ± 0.48 kJ mol^−1^, respectively. Contribution of S3 Thr-190 was also significant confirming its role in anchoring the ligand to the S3 binding site which was clear in the above conformational analysis (Fig. 7D). On the other hand, superior contribution was depicted for the catalytic His-41 rather than Cys-145 S1′ residues. Except for the S1 Glu-166 residue, the rest S1 and S1′ key binding residues exhibited minimal or even limited residue-wise energy contribution suggesting their insignificant role for the stability binding of the N3–Mpro complex.

### 4.3. *In vitro* results

#### 4.3.1. SARS-CoV-2 cell based inhibitory assay

**Fig. 10:**
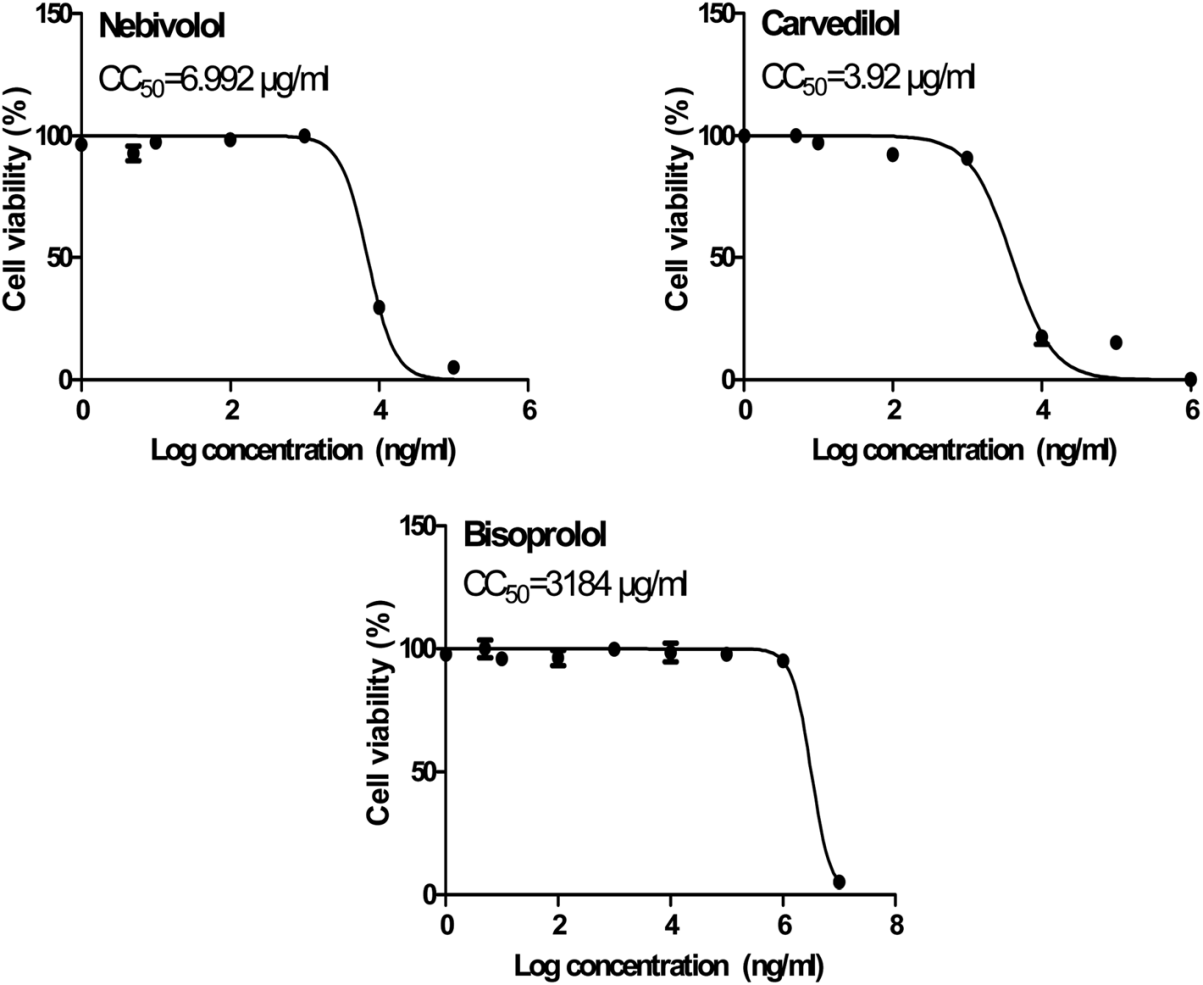
Cytotoxicity concentration 50 (CC_50_) values for the tested β-blockers (BIS, CAR, and NEB) on Vero E6 cells using nonlinear regression analysis of GraphPad Prism software (version 5.01) by plotting log cell viability *versus* normalized response (variable slope).

The obtained results of the cytotoxicity CC_50_ for the selected examined compounds (BIS, CAR, and NEB) on Vero E6 cells (Fig. 10) supplied us with the safe concentrations for each tested compound on the cells to be used in other *in vitro* tests.

**Fig. 11:**
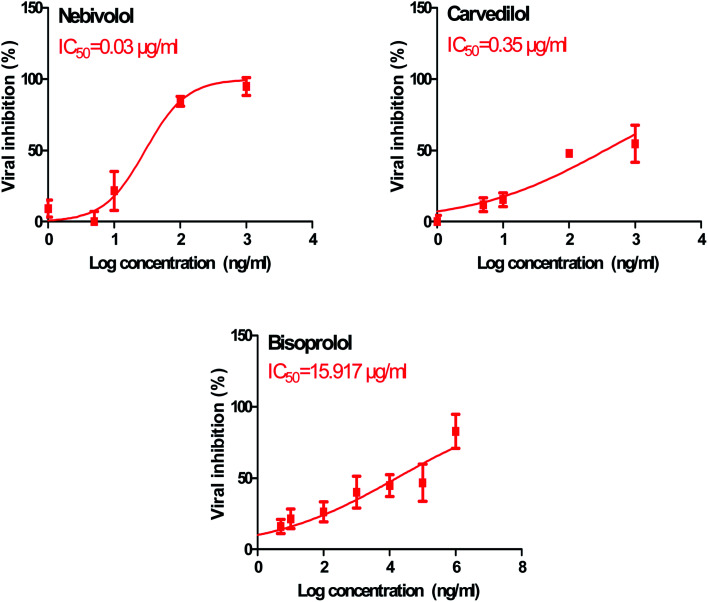
Inhibitory concentration 50 (IC_50_) of the tested β-blockers (BIS, CAR, and NEB): antiviral activity against Severe Acute Respiratory Syndrome Coronavirus 2 (SARS-CoV-2) (hCoV-19/Egypt/NRC-03/2020) (Accession Number on GSAID: EPI_ISL_430820) Vero E6 cells using nonlinear regression analysis of GraphPad Prism software (version 5.01) by plotting log inhibitory *versus* normalized response (variable slope).

In order to calculate the dose required to inhibit 50% of the pathogenic SARS-CoV-2, the inhibitory concentration 50 (IC_50_) for each examined compound was determined (Fig. 11). It is worth mentioning that the obtained IC_50_ values for the tested β-blockers were very promising and compatible with their recommended affinity order and predicted intrinsic activities from the early molecular docking studies which were also confirmed by the molecular dynamics simulations. Especially NEB achieved the best potential anti-SARS-CoV-2 activity with IC_50_ equals 0.030 μg ml^−1^. Also, its selectivity index was found to be 233.066. On the other hand, CAR was also found to have a promising inhibitory activity against SARS-CoV-2 with IC_50_ equals 0.350 μg ml^−1^ and with a selectivity index of 11.200. Moreover, the IC_50_ value of BIS was found to be15.917 μg ml^−1^ and its selectivity index was calculated as 200.037. The collective CC_50_ and IC_50_ values of the tested compounds together with their corresponding selectivity indexes were presented in Table 5.

**Table 5:** Cytotoxicity and SARS-CoV-2 inhibitory effects of the examined β-blockers (BIS,CAR, and NEB), besides their selectivity indexes

No.	Name	CC_50_ (μg ml^−1^)	IC_50_ (μg ml^−1^)	Selectivity index CC_50_/IC_50_
1	Bisoprolol	3184	15.917	200.037
2	Carvedilol	3.920	0.350	11.200
3	Nebivolol	6.992	0.030	233.066

The aforementioned *in vitro* results confirm greatly our proposed rational concerning the predicted anti-SARS-CoV-2 activity for the β-adrenergic blockers containing ethanolamine moieties. Furthermore, these results approve our computational findings through molecular docking and molecular dynamics which consequently suggest the strong expected activities for the other studied and discussed β-adrenergic blocker members as well. Accordingly, this gives us a clear idea for the promising expected β-adrenergic blockers activities against SARS-CoV-2 and these activities may be enhanced after further chemical structures' modification. So, we can deal with them as promising lead compounds for further modifications which requires also the study of their structure–activity relationships (SAR).

#### 4.3.2. SARS-CoV-2 main protease assay

**Fig. 12:**
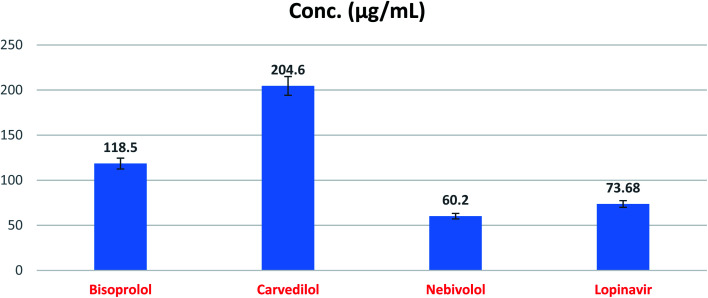
SARS-CoV-2 Mpro inhibitory concentration 50 (IC_50_) of the tested β-blockers (BIS, CAR, and NEB).

The expected SARS-CoV-2 Mpro inhibitory effects of the three tested compounds (BIS, CAR, and NEB) towards the SARS-CoV-2 Mpro enzyme were confirmed utililzing SARS-CoV-2 Mpro assay. The obtained results showed very promising SARS-CoV-2 Mpro inhibitory activities for BIS, CAR, and NEB (IC_50_ = 118.50, 204.60, and 60.20 μg ml^−1^, respectively) compared to lopinavir (IC_50_ = 73.68 μg ml^−1^) as a reference standard (Fig. 12 and Table 6). It is worth mentioning that NEB achieved the superior SARS-CoV-2 Mpro inhibitory activity which exceeds that of the reference standard (lopinavir) by approximately 0.82 fold.

**Table 6:** SARS-CoV-2 Mpro inhibitory effects of the examined β-blockers (BIS,CAR, and NEB)

No.	Name	IC_50_ (μg ml^−1^)	SD±
1	Bisoprolol	118.50	6.01
2	Carvedilol	204.60	10.40
3	Nebivolol	60.20	3.05
4	Lopinavir	73.68	3.74

### 4.4. Structure–activity relationship (SAR) studies

**Fig. 13:**
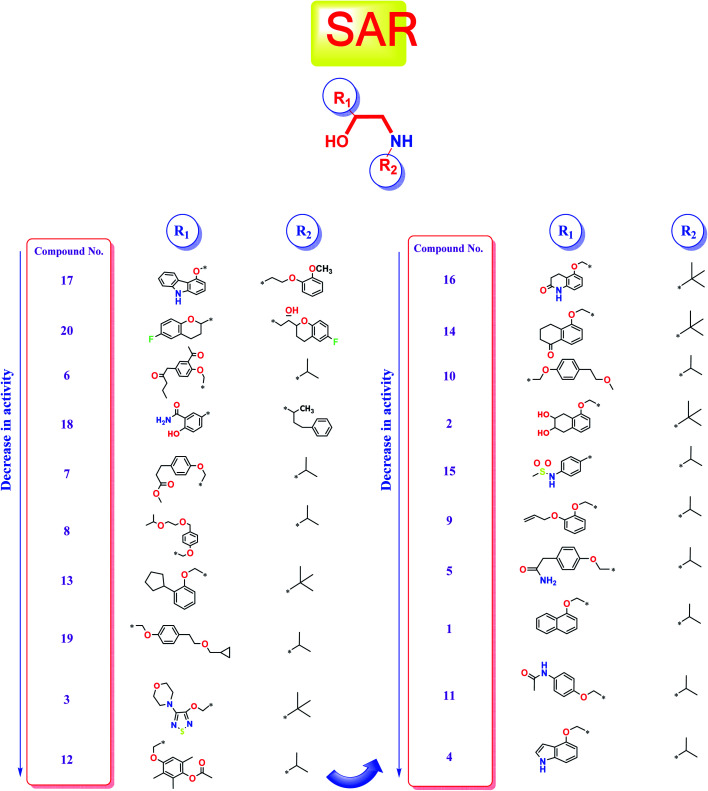
Structure–Activity Relationship (SAR) studies of the FDA-approved β-adrenergic blockers (1–20) containing ethanolamine moieties.

Studying the structure–activity relationship of tested β-adrenergic blockers according to their binding affinities to SARS-CoV-2 Mpro showed the following results (Fig. 13):

(a) Among tested β-adrenergic blockers and based on the docking results, the best activity against Covid-19 virus Mpro is accomplished when aryloxypropanolamine scaffold is used where the aryl group structure is 3-(9*H*-carbazol-4-yloxy) (R_1_) and the amino-terminal (R_2_) is (2-methoxyphenoxy) ethylamine derivative, (compound 17, Carvedilol).

(b) Additionally, the studied SAR revealed that the replacement of aryl group (R_1_) with 6-fluoro benzopyran or 3-acetyl phenyl butanamide and amino terminal (R_2_) with [6-fluoro benzopyran] hydroxy ethyl or isopropyl, (compounds 20 and 6), respectively, granted favorable conserved activity against the viral Mpro.

(c) Moreover, the studied SAR showed that R_1_ may be conformationally rigid structures (compounds 17, 18, and 20) or flexible structures (compounds 6, 7, and 8) with different binding interaction with SARS-CoV-2 Mpro amino acids hence ensuring the feasible stability of these compounds in the viral Mpro pocket.

(d) Collectively, different substitutions on both sides of ethanolamine moiety of β-adrenergic blockers (R1 and R2) affect greatly their binding affinities inside the Mpro cavity of SARS-CoV-2 as depicted in detail in Fig. 13.

(e) The better activities of CAR and NEB may be attributed to the favorable hydrophobic interactions between the N-terminal attached aromatic moieties in both compounds with the essential amino acid residues forming the hydrophobic cleft of SARS-CoV-2 Mpro pocket (His-41in case of CAR and Met-165 in case of NEB) which are missing in all the lower active ones.

## 5. Conclusion

The present study suggests the potential anti-SARS-CoV-2 activities of twenty β-adrenergic blockers containing the hydroxyethylamine and hydroxyethylene moieties. Three members revealed the best *in silico* results (Carvedilol (CAR), Nebivolol (NEB), and Bisoprolol (BIS)) and hence, were subjected to further *in vitro* testing for their inhibitory activities against SARS-CoV-2 (IC_50_ values were found to be 0.030, 0.350, and 15.917 μg ml^−1^, respectively). Also, the SARS-CoV-2 Mpro inhibitory effects of the tested compounds (BIS, CAR, and NEB) were evaluated and the results showed very promising inhibitory activities (IC_50_ = 118.50, 204.60, and 60.20 μg ml^−1^, respectively) compared to lopinavir (IC_50_ = 73.68 μg ml^−1^) as a reference standard. These results greatly confirmed our proposed rational and agreed with our computational findings through molecular docking (targeting the viral Mpro) and molecular dynamics which consequently suggest the strong expected activities for the other studied and discussed β-adrenergic blockers as well. So, we recommend further preclinical and clinical studies for the fast repurposing of the already found and discussed FDA-approved β-blockers as proposed candidates for the management of Covid-19 viral pandemic. Additionally, the studied medications can deal with as promising lead compounds for further structural modifications especially after shedding light on their structure–activity relationships (SAR) to enhance their activity against SARS-CoV-2. Finally, CAR and NEB are greatly recommended for fast drug repurposing or even structural modification in order to get a potential therapeutic effectively targeting SARS-CoV-2. Also, based on the aforementioned results, we may recommend the use of β-blockers for hypertensive COVID-19 patients by regulating high blood pressure and decreasing the cellular entry of SARS-CoV-2 but in lower doses for normal blood pressure COVID-19 patients.

## Author contributions

Conceptualization: Mohammed I. A. Hamed, Ayman Abo Elmaaty, and Ahmed A. Al-karmalawy; data curation: Khaled M. Darwish, Ahmed Mostafa, Ayman Abo Elmaaty, and Ahmed A. Al-karmalawy; formal analysis: Khaled M. Darwish, Ahmed Mostafa, Ayman Abo Elmaaty, and Ahmed A. Al-karmalawy; funding acquisition: Raya Soltane, Amani Chrouda, and Ahmed A. Al-karmalawy; investigation: Ayman Abo Elmaaty and Ahmed A. Al-karmalawy; methodology: Khaled M. Darwish, Sameh S. Elhady, Ahmed Mostafa, Noura M. Abo Shama, Ahmed E.Khodir, Ayman Abo Elmaaty, and Ahmed A. Al-karmalawy; project administration: Ayman Abo Elmaaty and Ahmed A. Al-karmalawy; resources: Raya Soltane and Ahmed A. Al-karmalawy; software: Khaled M. Darwish, Ayman Abo Elmaaty, and Ahmed A. Al-karmalawy; supervision: Ahmed A. Al-karmalawy; validation: Khaled M. Darwish and Ahmed A. Al-karmalawy; visualization: Khaled M. Darwish, Ahmed Mostafa, Ayman Abo Elmaaty, and Ahmed A. Al-karmalawy; writing – original draft: Mohammed I. A. Hamed, Raya Soltane, Amani Chrouda, Ayman Abo Elmaaty, and Ahmed A. Al-karmalawy; writing – review & editing: Hamada S. Abulkhair, Ayman Abo Elmaaty, and Ahmed A. Al-karmalawy. All authors approved the final version of the manuscript.

## Conflicts of interest

The authors declare no conflict of interest.

## Supplementary Material

RA-011-D1RA04820A-s001
