# Supplementary material for: β-Blockers bearing hydroxyethylamine and hydroxyethylene as potential SARS-CoV-2 Mpro inhibitors: rational based design, in silico, in vitro, and SAR studies for lead optimization
Source: RSC Adv. 2021 Nov 3;11(56):35536–58. doi: 10.1039/d1ra04820a (PMC9043270; doi:10.1039/d1ra04820a)
Supplement: RA-011-D1RA04820A-s001 [file RA-011-D1RA04820A-s001.pdf]

Electronic Supplementary Material (ESI) for RSC Advances.
This journal is © The Royal Society of Chemistry 2021

β-Blockers bearing Hydroxyethylamine and Hydroxyethylene as Potential SARS-CoV-2 Mpro Inhibitors: Rational based Design, *In Silico*, *In Vitro*, and SAR Studies for Lead Optimization

Mohammed I. A. Hamed[1,ψ], Khaled M. Darwish[2,ψ], Raya Soltane[3,4,ψ], Amani Chrouda[5,6,7], Ahmed Mostafa[8], Noura M. Abo Shama[8], Sameh S. Elhady[9], Hamada S. Abulkhair[10,11], Ahmed E.Khodir[12], Ayman Abo Elmaaty[13,*], and Ahmed A. Al-karmalawy[11,*]

[1] Department of Organic and Medicinal Chemistry, Faculty of Pharmacy, Fayoum University, Fayoum 63514, Egypt. Email: mia06@fayoum.edu.eg
[2] Department of Medicinal Chemistry, Faculty of Pharmacy, Suez Canal University, Ismailia 41522, Egypt. Email: khaled_darwish@pharm.suez.edu.eg

[3] Department of Basic Sciences, Adham University College, Umm Al-Qura University, Saudi Arabia; Email: rasoltan@uqu.edu.sa

[4] Faculty of Sciences, Tunis El Manar University, Tunisia.

[5] Department of Chemistry, College of Science Al-Zulfi, Majmaah University, Al-Majmaah 11952, Saudi Arabia; Email: amain.c@mu.edu.sa

[6] Laboratory of Interfaces and Advanced Materials, Faculty of Sciences, Monastir University, Monastir 5000, Tunisia.

[7] Institute of Analytical Sciences, UMR CNRS-UCBL-ENS 5280, 5 Rue la Doua, 69100 Villeurbanne, CEDEX, France.

[8] Center of Scientific Excellence for Influenza Viruses, National Research Centre, Dokki, 12622, Cairo, Egypt; Email: ahmed_elsayed@daad-alumni.de and noura.mahrous1995@gmail.com

[9] Department of Natural Products, Faculty of Pharmacy, King Abdulaziz University, Jeddah 21589, Saudi Arabia. Email: ssahmed@kau.edu.sa

[10] Department of Pharmaceutical Organic Chemistry, Faculty of Pharmacy (Boys), Al-Azhar University, Nasr City 11884, Cairo, Egypt. hamadaorganic@azhar.edu.eg

[11] Department of Pharmaceutical Chemistry, Faculty of Pharmacy, Horus University-Egypt, New Damietta 34518, Egypt. Email: akarmalawy@horus.edu.eg

[12] Department of Pharmacology, Faculty of Pharmacy, Horus University-Egypt, New Damietta, 34518, Egypt. Email: akhodir@horus.edu.eg

[13] Department of Medicinal Chemistry, Faculty of Pharmacy, Port Said University, Port Said 42526, Egypt. Email: ayman.mohamed@pharm.psu.edu.eg

*Corresponding authors:

Ahmed A. Al-Karmalawy: Email: akarmalawy@horus.edu.eg

ORCID: 0000-0002-8173-6073

Ayman Abo Elmaaty: Email: ayman.mohamed@pharm.psu.edu.eg

ORCID: 0000-0002-4634-3039

ψ These authors equally contributed to this work.

Figure SI1: 2D and 3D pictures of the binding interactions of the examined FDA-approved *β*-adrenergic blockers (**1-20**) within SARS-CoV-2 Mpro pocket (PDB: 6LU7) compared to the N3 inhibitor (**21**, Docked). Red and gray dashed lines (in 3D pictures) refer to hydrogen bonds and hydrophobic interactions, respectively.

No.	*β*-adrenergic blockers	2D pictures	3D pictures
**1**	Propranolol	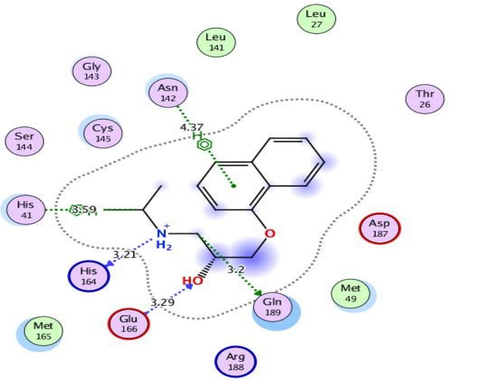	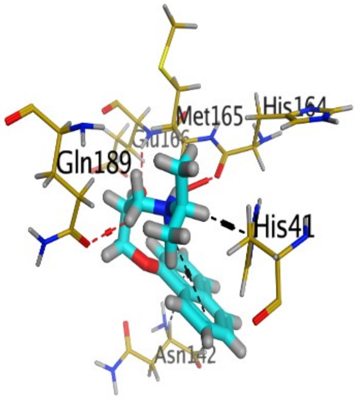
**2**	Nadolol	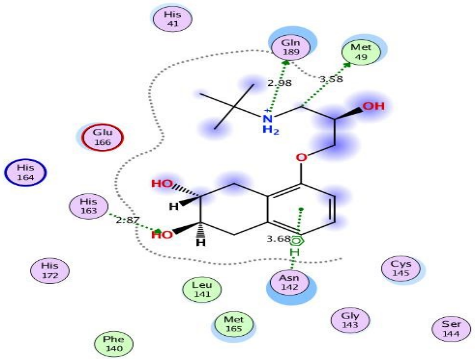	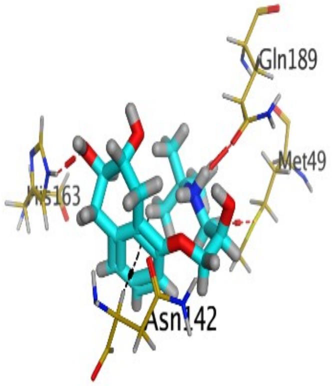

<table>
<tr>
<td>3</td>
<td>Timolol</td>
<td>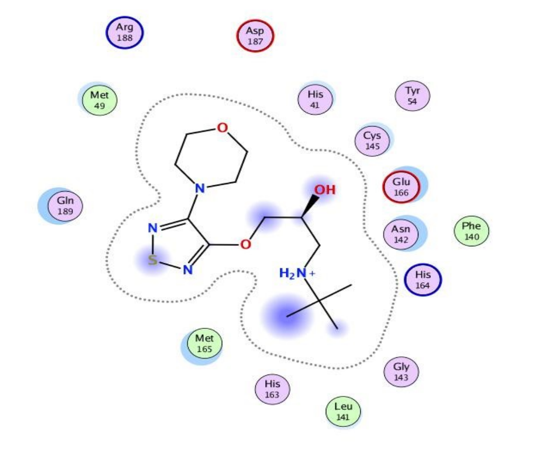
</td>
<td>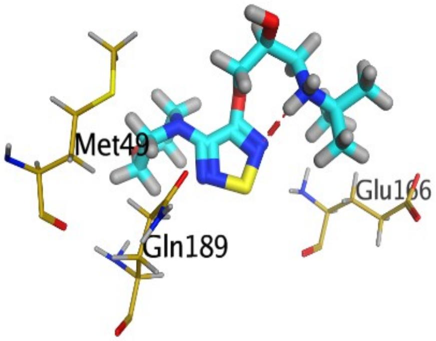
</td>
</tr>
<tr>
<td>4</td>
<td>Pindolol</td>
<td>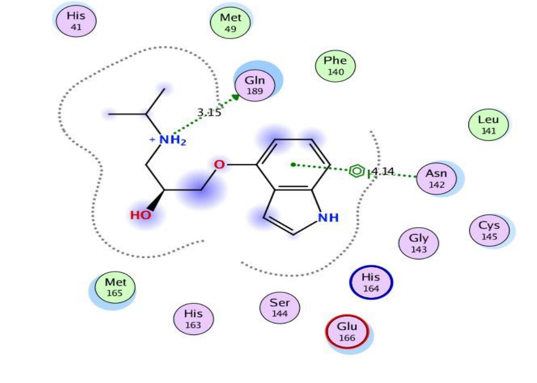
</td>
<td>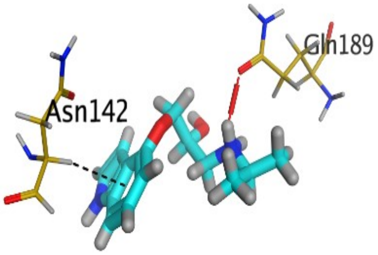
</td>
</tr>
<tr>
<td>5</td>
<td>Atenolol</td>
<td>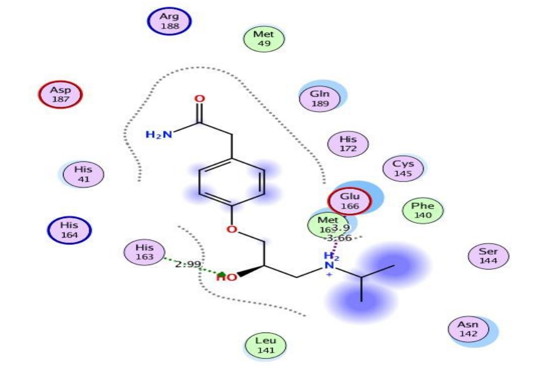
</td>
<td>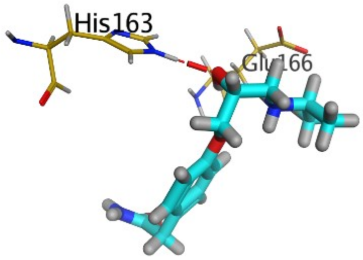
</td>
</tr>
<tr>
<td>6</td>
<td>Acebutolol</td>
<td>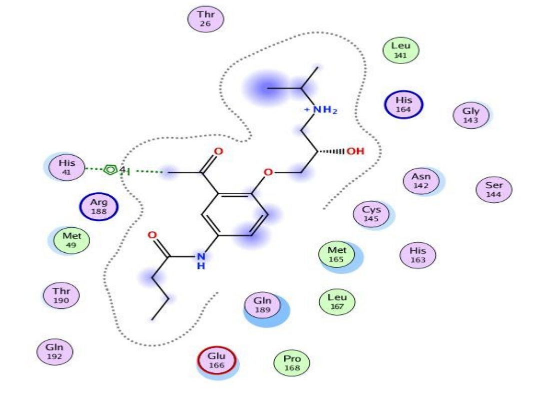
</td>
<td>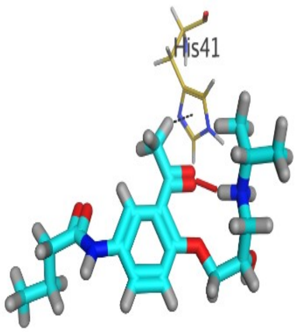
</td>
</tr>
</table>

**7**	Esmolol	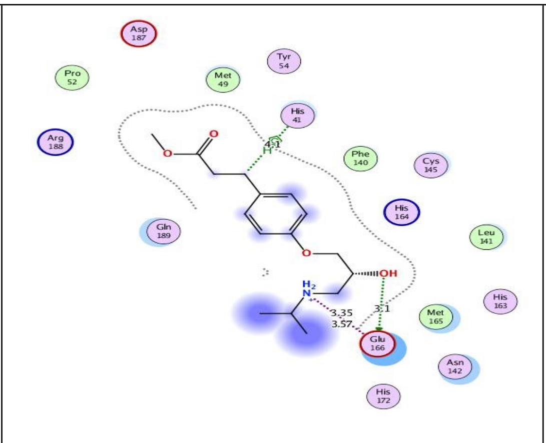	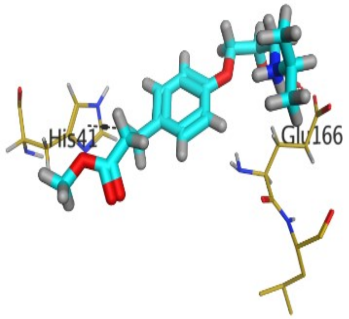
**8**	Bisoprolol	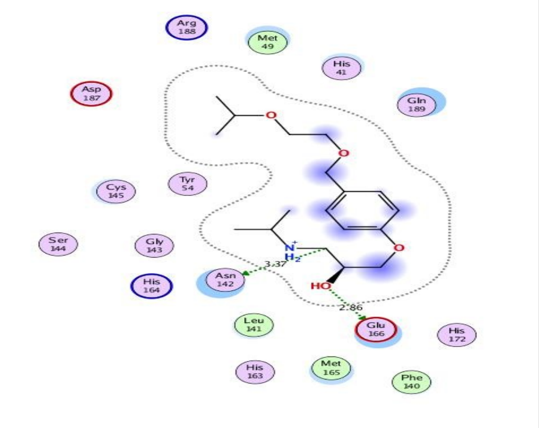	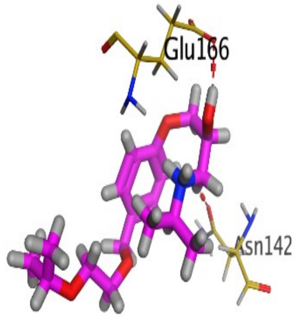
**9**	Oxprenolol	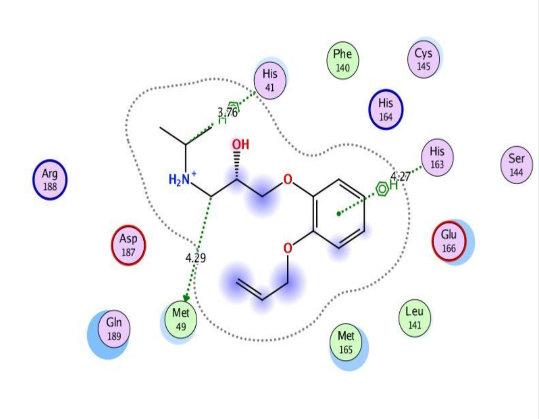	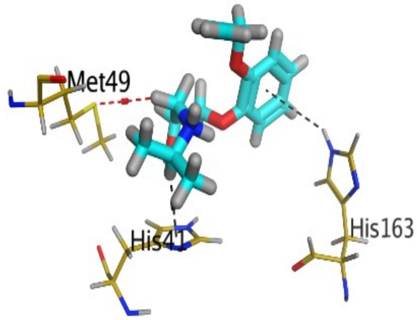
**10**	Metoprolol	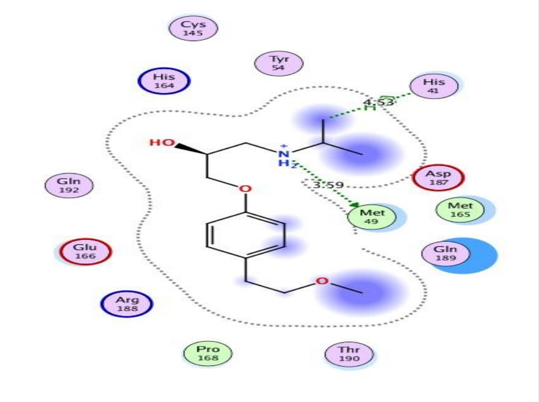	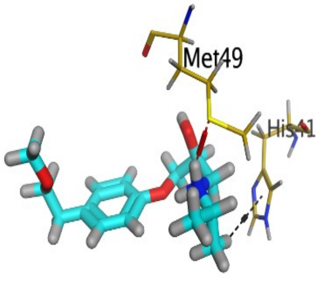

**11**	Practolol	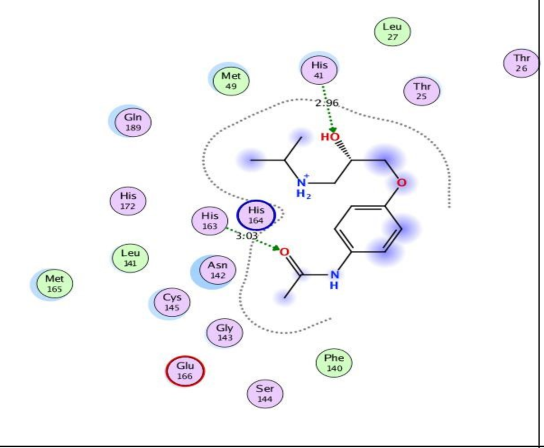	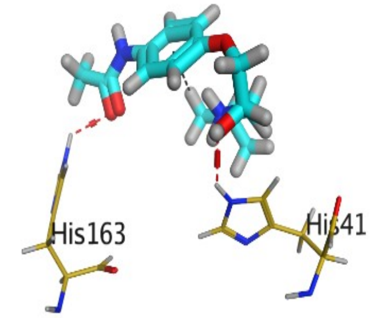
**12**	Metipranolol	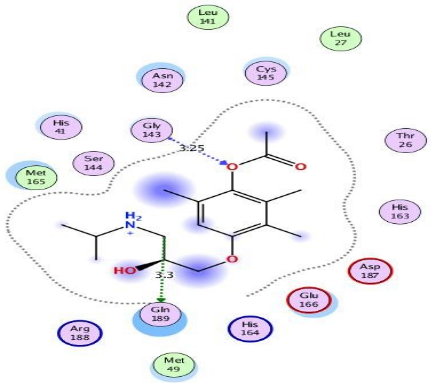	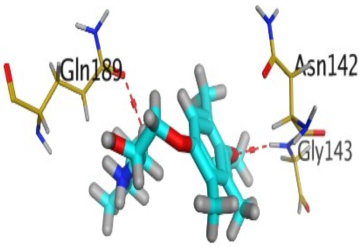
**13**	Penbutolol	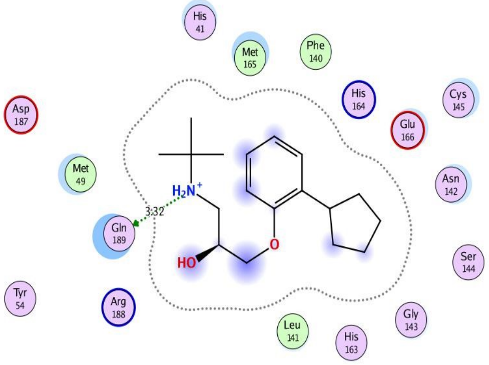	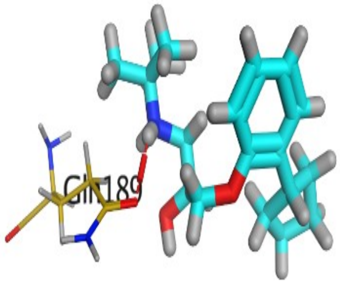

**14**	Levobunolol	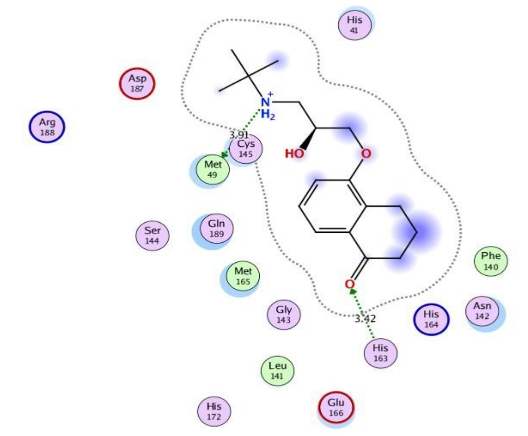	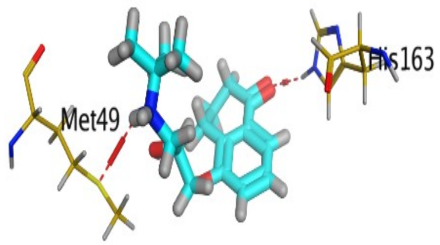
**15**	Sotalol	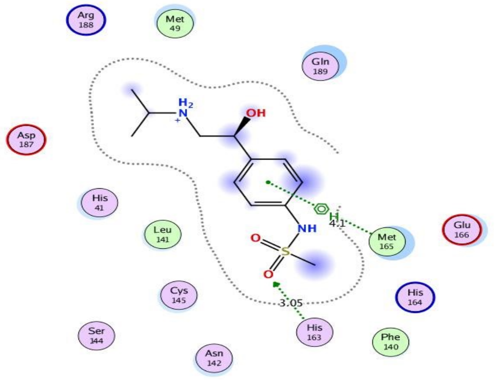	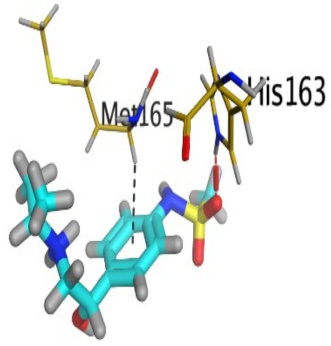
**16**	Carteolol	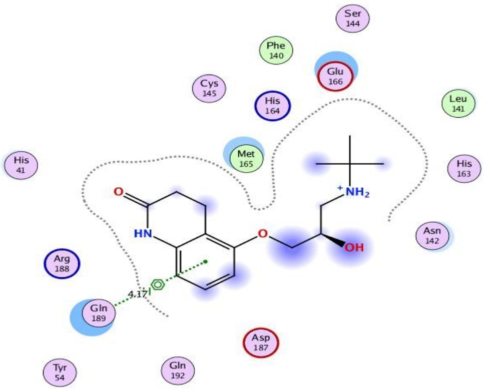	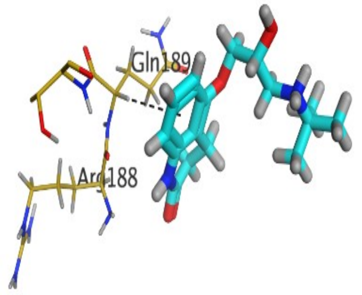

<table>
<tr><td>17</td><td>Carvedilol</td><td>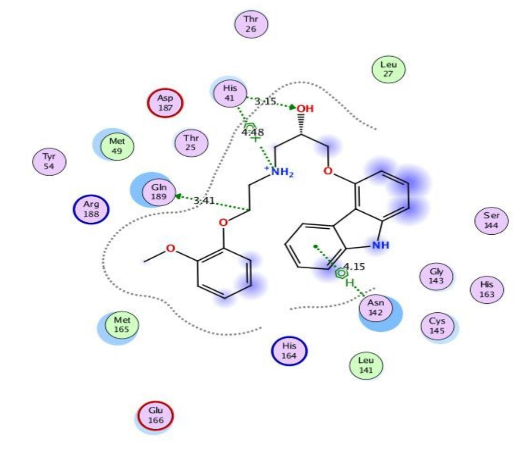
</td><td>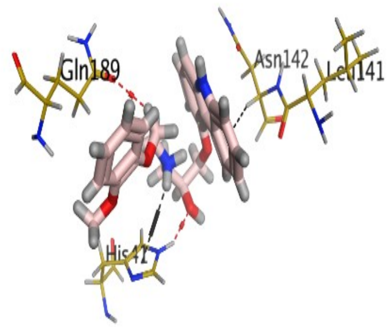
</td></tr>
<tr><td>18</td><td>Labetalol</td><td>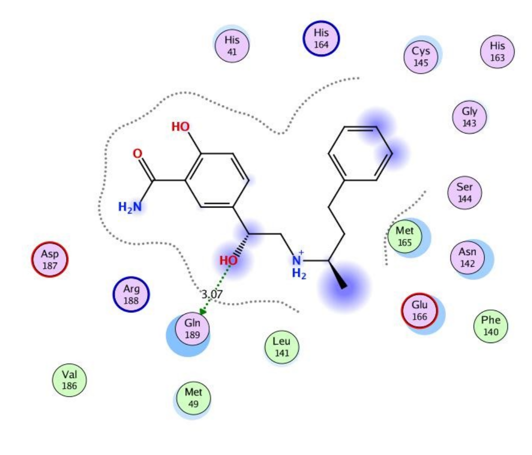
</td><td>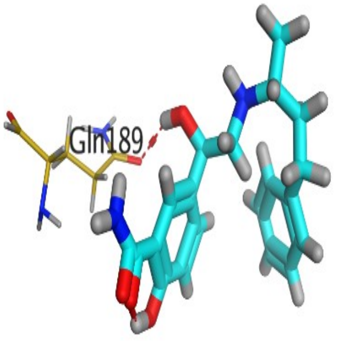
</td></tr>
<tr><td>19</td><td>Betaxolol</td><td>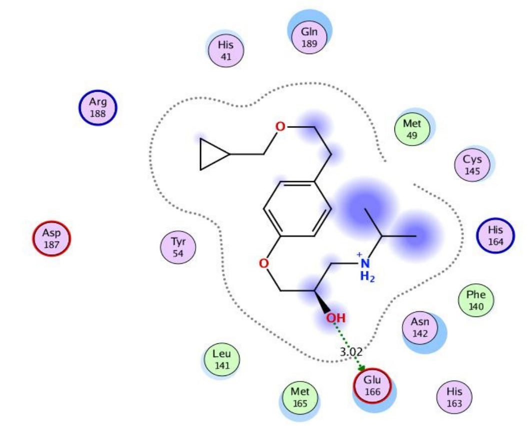
</td><td>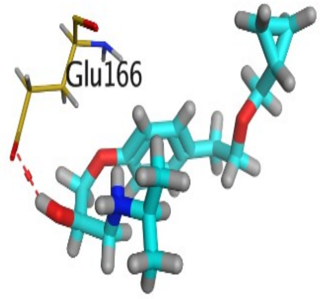
</td></tr>
</table>

**20**	Nebivolol	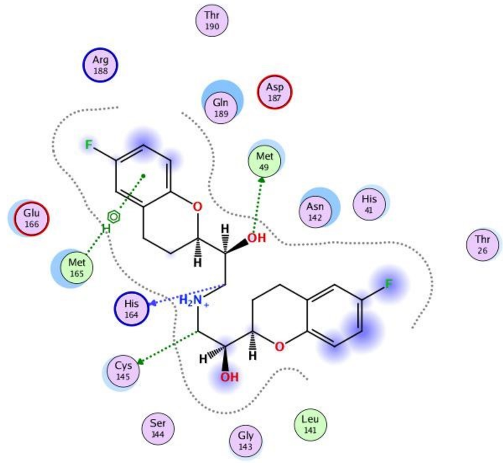	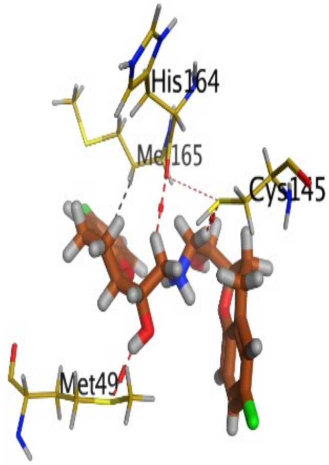
**21**	N3	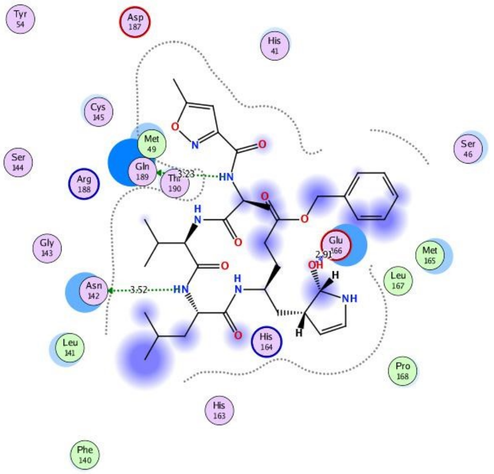	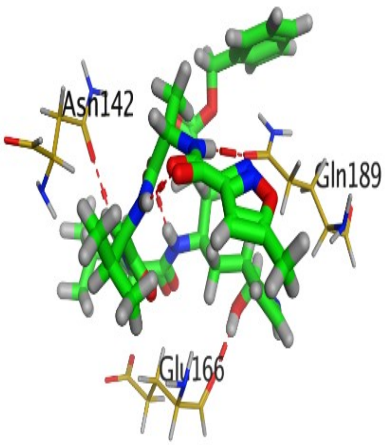

Table SI1: 3CL Protease (SARS-CoV-2) Assay Results:

Researcher	: Dr. Ahmed Al-karmalawy email: ahmed.alkarmalawy2019@gmail.com mob. 01092147330
Assay	: COV-3CL protease assay
Samples	: 03 compounds.
Cell line	: ---
Reference	: ---
Date	: 19/08/2021

Lab Report

ser	Compound		Results	
	code	MW g/mol	COV-3CL protease IC50 ug/ml	SD ±
1	Nebivolol (HCQ)		60.2	3.05
2	Carvedilol		204.6	10.4
3	Bisoprolol (fumareite)		118.5	6.01
***	**Lopinavir**		73.68	3.74

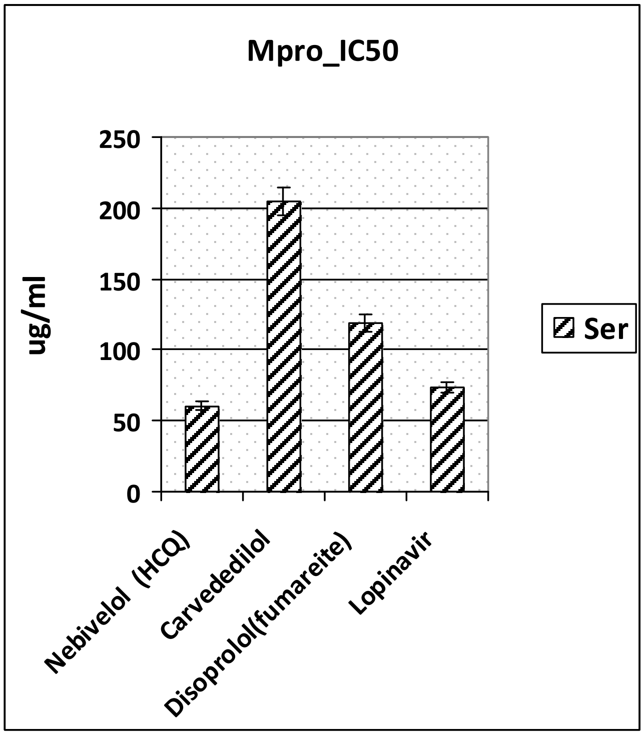

Detailed results

3CL

code	IC50	conc	log	%inh	T2	T1	ΔT	RFU2	RFU1	ΔRFU	slope	K.Activity	EC
Nebivolol (HCQ)		1000	3	75.19	30	0	30	24.81	0	24.81	3.3333	29.7723	120
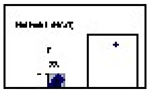		100	2	60.12	30	0	30	39.88	0	39.88	3.3333	47.8565	120
		10	1	28.46	30	0	30	71.54	0	71.54	3.3333	85.8489	120
		1	0	11.24	30	0	30	88.76	0	88.76	3.3333	106.513	120
EC				0	30	0	30	100	0	100	3.3333	120	120

code	IC50	conc	log	%inh	T2	T1	ΔT	RFU2	RFU1	ΔRFU	slope	K.Activity	EC
Carvedilol		1000	3	68.98	30	0	30	31.02	0	31.02	3.3333	37.2244	120
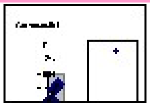		100	2	42.08	30	0	30	57.92	0	57.92	3.3333	69.5047	120
		10	1	11.86	30	0	30	88.14	0	88.14	3.3333	105.769	120
		1	0	5.739	30	0	30	94.26	0	94.26	3.3333	113.113	120
EC				0	30	0	30	100	0	100	3.3333	120	120

code	IC50	conc	log	%inh	T2	T1	ΔT	RFU2	RFU1	ΔRFU	slope	K.Activity	EC
Bisoprolol (fumareite)		1000	3	74.34	30	0	30	25.66	0	25.66	3.3333	30.7923	120
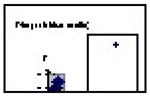		100	2	44.59	30	0	30	55.41	0	55.41	3.3333	66.4927	120
		10	1	21.87	30	0	30	78.13	0	78.13	3.3333	93.7569	120
		1	0	8.979	30	0	30	91.02	0	91.02	3.3333	109.225	120
EC				0	30	0	30	100	0	100	3.3333	120	120

code	IC50	conc	log	%inh	T2	T1	ΔT	RFU2	RFU1	ΔRFU	slope	K.Activity	EC
Lopinavir		1000	3	80.37	30	0	30	19.63	0	19.63	3.3333	23.5562	120
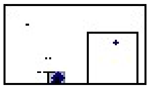		100	2	51.48	30	0	30	48.52	0	48.52	3.3333	58.2246	120
		10	1	23.89	30	0	30	76.11	0	76.11	3.3333	91.3329	120
		1	0	8.549	30	0	30	91.45	0	91.45	3.3333	109.741	120
EC				0	30	0	30	100	0	100	3.3333	120	120

**Nebivelol (HCQ)**	y = 22.351x + 10.225
**Carvededilol**	y = 21.994x - 0.827
**Disoprolol(fumareite)**	y = 21.88x + 4.624
**Lopinavir**	y = 24.305x + 4.614

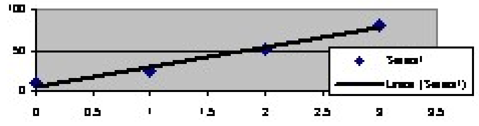

SI1: Methodology and protocol of 3CL Protease (SARS-CoV-2) Assay:

The *3CL Protease Assay Kit* is designed to measure 3CL Protease activity for screening and profiling applications, in a homogeneous assay with no time-consuming washing steps. The kit comes in a convenient 96-well format, with purified 3CL Protease, fluorogenic substrate, and 3CL Protease assay buffer for 100 enzyme reactions. 3CL inhibitor GC376 is also included as a positive control.

Protocol

Add **0.5 M DTT** to **3CL Protease Assay Buffer** so final DTT concentration is 1 mM. For example, add 10 µl of **0.5 M DTT** to 5 ml assay buffer. (DTT should be added just before use. Prepare only enough DTT-containing buffer as required for the assay. Store the remaining assay buffer at -20°C).

2) Thaw **3CL Protease** on ice. Upon first thaw, briefly spin tube containing enzyme to recover the full content of the tube. Aliquot **3CL Protease** into single use aliquots. Store remaining undiluted enzyme in aliquots at -80°C. Note: **3CL Protease** enzyme is sensitive to freeze/thaw cycles. Do not re-use diluted enzyme.

3) Dilute **3CL Protease** in **Assay buffer** (with 1 mM DTT) at 3-5 ng/µl (90-150 ng per reaction).

4) Add 30 µl **diluted 3CL Protease** enzyme solution to wells designated as "Positive Control", "Inhibitor Control" and "Test Sample". Add 30 µl **Assay buffer** (with 1 mM DTT) to the "Blank" wells.

**Component**	**Positive Control**	**Test Sample**	**Inhibitor Control**	**Blank**
3CL Protease (3-5 ng/µl)	30 µl	30 µl	30 µl	–
Assay Buffer (with DTT)	–	–	–	30 µl
GC376 (500 µM)	–	–	10 µl	–
Test Inhibitor	–	10 µl	–	–
Inhibitor Buffer (no inhibitor)	10 µl	–	–	10 µl
Substrate solution	10 µl	10 µl	10 µl	10 µl
**Total**	**50 µl**		**50 µl**	**50 µl**

5) Dilute 50 µg **GC376** in 200 µl water to obtain a 500 µM solution. Aliquot and store remaining solution in aliquots at -80°C. Add 10 µl **GC376** (500 µM) to the wells labeled "Inhibitor Control".

6) Prepare the inhibitor solution.

The final concentration of DMSO in the assay should not exceed 1%. If the inhibitor compound is dissolved in DMSO, make a 100-fold higher concentration of the compound than the highest concentration you want to test in DMSO. Then make a 20-fold dilution in 1X assay buffer (at this step the compound concentration is 5-fold higher than the final concentration).

If the inhibitor compound is dissolved in water, make a solution of the compound 5-fold higher than the final concentration in 3CL Protease assay buffer (with 1 mM DTT). For example, diluting 50 µg GC376 in 200 µl water (step 5) creates a 500 µM solution. Adding 10 ul to the assay (final volume 50 µl) results in a 100 µM final concentration.

7) Add 10 µl inhibitor to each well designated "Test Sample". Add 10 µl 1X assay buffer or 5% DMSO (depending on which inhibitor solution is used) to "Blank" and "Positive Control" wells.

8) Preincubate enzyme with the inhibitor for 30 min at room temperature with slow shaking.

9) Dilute 5 mM **3CL Protease substrate** 1:20 in assay buffer with DTT, to make a 250 µM solution. Dilute only enough as is required for the assay.

10) Start reaction by adding 10 µl of the substrate solution to each well (Final concentration of the **3CL Protease substrate** in a 50 µl reaction is 50 µM).

11) Incubate at room temperature for overnight. Seal the plate with the plate sealer. Measure the fluorescence intensity in a microtiter plate-reading fluorimeter capable of excitation at a wavelength 360 nm and detection of emission at a wavelength 460 nm. The fluorescence intensity can also be measured kinetically. "Blank" value is subtracted from all other values.
